# Single-cell RNA cap and tail sequencing (scRCAT-seq) reveals subtype-specific isoforms differing in transcript demarcation

**DOI:** 10.1038/s41467-020-18976-7

**Published:** 2020-10-13

**Authors:** Youjin Hu, Jiawei Zhong, Yuhua Xiao, Zheng Xing, Katherine Sheu, Shuxin Fan, Qin An, Yuanhui Qiu, Yingfeng Zheng, Xialin Liu, Guoping Fan, Yizhi Liu

**Affiliations:** 1 State Key Laboratory of Ophthalmology, Zhongshan Ophthalmic Center, Sun Yat-Sen University, Guangzhou, China; 2 Earth, Planetary and Space Sciences, UCLA, Los Angeles, CA USA; 3 Medical Scientist Training Program, David Geffen School of Medicine, UCLA, Los Angeles, CA USA; 4 Department of Human Genetics, David Geffen School of Medicine, UCLA, Los Angeles, CA USA

**Keywords:** Gene expression, Transcriptomics

## Abstract

The differences in transcription start sites (TSS) and transcription end sites (TES) among gene isoforms can affect the stability, localization, and translation efficiency of mRNA. Gene isoforms allow a single gene diverse functions across different cell types, and isoform dynamics allow different functions over time. However, methods to efficiently identify and quantify RNA isoforms genome-wide in single cells are still lacking. Here, we introduce single cell RNA Cap And Tail sequencing (scRCAT-seq), a method to demarcate the boundaries of isoforms based on short-read sequencing, with higher efficiency and lower cost than existing long-read sequencing methods. In conjunction with machine learning algorithms, scRCAT-seq demarcates RNA transcripts with unprecedented accuracy. We identified hundreds of previously uncharacterized transcripts and thousands of alternative transcripts for known genes, revealed cell-type specific isoforms for various cell types across different species, and generated a cell atlas of isoform dynamics during the development of retinal cones.

## Abstract (web summary)

Most single-cell RNA sequencing methods have limited ability to profile the transcriptome at isoform resolution. Here the authors develop scRCAT-seq to characterize the 5′- and 3′-ends of transcripts in single cells, and identify cell-type specific alternative transcript isoforms.

## Introduction

The extent of cellular heterogeneity across different tissues and cell types has become increasingly apparent with the development of genomics technology, especially single-cell omics sequencing^1–3^. With the launch of initiatives such as the Human Cell Atlas^4,5^, the regulatory mechanisms behind cell-specific gene transcription have gained increasing attention, including both transcript abundance and alternative isoform usage^6,7^. RNA isoform variability includes intron inclusion, exon skipping, and alternative choice of transcription start sites (TSSs)^8^ and transcription end sites (TESs)^9,10^. Alternative TSSs and TESs, which can affect mRNA stability, translation, and localization^9–13^, are considered the principal drivers of transcript isoform diversity across tissues, and underlie the majority of isoform-mediated, cell-type-specific proteomes^14^.

Previous studies have demonstrated the widespread heterogeneity of transcript isoforms with alternative 5′-TSS or 3′-TES (also called alternative polyadenylation, APA) across different tissues, resulting in the discovery of new transcripts with tissue- or cell-type specificity, and allowing updates to transcript annotations of reference genome^12,15–17^. Despite considerable success in measurements made on bulk populations, current approaches for identifying RNA isoforms and the dynamics of TSS/TES choices in single cells are limited. Fundamentally, there is currently no genome-wide method for accurate, efficient, and quantitative analysis of RNA isoforms in single cells. Most single-cell transcriptome approaches are based on single-ended quantification of RNA molecules (5′ or 3′) which give partial information on one end but not the whole transcript^3,18,19^, resulting in loss of important information about the other end^12^. Methods based on single-cell full-length cDNA amplification such as Smart-seq2 can detect the full-length cDNA, but its coverage at both ends is low, and it is not possible to accurately distinguish the start and end positions of different transcript isoforms of the same gene^20,21^. Recently, approaches based on long-read RNA sequencing technologies can identify RNA isoforms of thousands of cells, but challenges still remain. For example, the current cost for genome-wide quantification is too high, and the requirement of several micrograms of cDNA input requires extensive PCR amplification from picograms of mRNA of a single cell, which inevitably results in higher PCR bias towards specific isoforms^12,17,22^.

In order to address these problems, here we introduce a simple and efficient approach based on well-established short-read sequencing platforms to explicitly exploit transcription initiation and termination sites for RNA isoforms in single cells. When deployed in conjunction with optimized machine learning models, scRCAT-seq is more accurate, cost-effective, and efficient than existing methods in profiling isoforms with alternative TSS/TES choices.

## Results

### The accuracy of scRCAT-seq

**Fig. 1:**
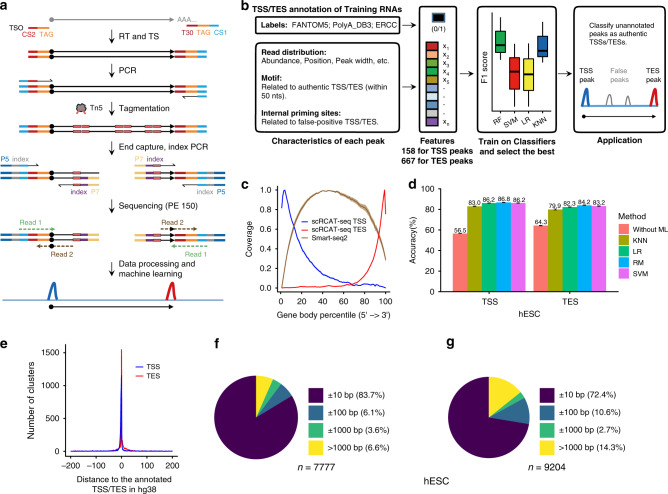
Overview of scRCAT-seq. **a** Schematic of the scRCAT-seq method. Full-length cDNA was synthesized by template-switching reverse transcription, amplified by PCR, and tagmented with Tn5 transposases. The TAG added to both ends contains the UMI (unique molecular identifier) and CI (cell identifier). Both 5′ and 3′ ends of the cDNA were captured and amplified by PCR, producing indexed libraries for pooled sequencing. Sequencing data were processed and transcription start sites (TSSs) and transcription end sites (TESs) were identified using machine learning models. CS1: common sequence 1; CS2: common sequence 2; TSO: Template-switching oligo; T30: 30 repeating T bases. **b** Schematic of the machine learning models. Features were collected based on characteristics related to the peaks, including the read distribution, motifs related to real TSSs/TESs, and sequence features related to internal false-positive signals, and used to train RF, LR, SVM, and KNN models. **c** Gene body coverage of scRCAT-seq reads derived from DRG (*n* = 18). Shown is the mean coverage of reads shaded by 95% confidence intervals. **d** Accuracy in identifying authentic TSSs and TESs with different machine learning models. Error bars represent standard deviation of the mean (*n* = 3). **e** Distance of the identified TSSs/TESs to those annotated in hg38. TSSs/TESs were identified from the scRCAT-seq peaks derived from hESC with the RF model. **f** Pie chart illustrating the distribution of the identified TSSs in hESC relative to the TSSs in the FANTOM5 database. The total number of TSS peaks identified after optimization by the machine learning models is indicated under the pie chart. **g** Pie chart illustrating the distribution of the identified TSSs in hESC relative to the TESs in PolyA_DB3. Source data are provided as a Source data file.

To develop scRCAT-seq, we adopted a strategy to capture the boundaries of transcripts at both 5′ and 3′ ends^23^. We first added a specific sequence tag (containing the UMI and cell barcode) to both ends of full-length cDNAs during reverse transcription and template switching, and further amplified the cDNAs of each single cell based on a modified Smart-seq2 protocol^21^. After tagmentation with Tn5 transposases, fragments containing the tags and single ends of the cDNA (either 5′ or 3′ end) were captured by targeted PCR, and cell barcodes (the same as sequencing indexes) were added to the libraries during amplification. Libraries were sequenced PE150 on standard Illumina sequencing platforms. To determine TSSs, we mapped the reads with the tag to the genome and obtained the mapping position of the fragments adjacent to the “GGG” added during template switching. To determine TESs, we mapped the reads with a poly-A tail to the genome, and obtained the mapping sites of the fragments adjacent to the poly A (Fig. 1a). Peaks were called using the CAGEr package^24^ and used to identify TSSs and TESs of transcripts. Either UMI (contained in the tag) counts or read counts were used to quantify the corresponding TSS/TES ends. The protocol takes less than two days from cell picking to having a final library ready for sequencing, and the cost for library construction was 28 dollars per single cell in China, similar to Smart-seq2.

We anticipated false-positive events based on previously reported false-positive TSSs/TESs, which resulted from factors such as RNA degradation during processing, internal priming and template-switching artifacts^25^ during reverse transcription, or DNA artifacts during PCR amplification. To increase the accuracy of mapping peaks to the TSSs/TESs of transcripts, we decided to classify the peaks into TRUE or FALSE groups of TSSs/TESs by employing machine learning algorithms (Fig. 1b). We generated three groups of features based on the following characteristics of the peaks: (1) The scRCAT-seq read distribution; (2) Location of the motifs which were associated with real TSSs/TESs around the peaks; (3) Sequence motifs possibly resulting in false-positive TSS/TES, such as the internal priming sites (see “Methods”) (Supplementary Table 1). We implemented four widely used machine learning models: logistic regression classifier (LR), random forest (RF), and support vector machine (Gaussian kernel SVM), and k-nearest neighbor (KNN). Performance was assessed using accuracy on the spike-in RNA ERCC transcripts, which has a ground truth. As expected, the majority of the reads with tags were distributed at the terminal sides of transcripts (Fig. 1c), though some appeared in the middle of the transcripts, which contribute to the false-positive peaks (Supplementary Fig. 1a).

The machine learning models significantly improved the accuracy, and of the four tested models, RF showed the best performance, improving the accuracy by 2.5- (38.3% versus 96.9%) and 3.9-fold (25.2% versus 99.6%) for TSS and TES, respectively (Supplementary Fig. 1b), with sequencing depth of 4 million reads per sample (Supplementary Table 2). Similarly, looking into ERCC data generated by other methods^19,26^, such as C1 CAGE, C1 STRT, we also found high false-positive rates for peaks identified as TSSs in these datasets (Supplementary Fig. 1c), and applying the machine learning model increased the accuracy to above 88.9% (Supplementary Fig. 1d), indicating that our model can also be applied to other datasets that contain high false-positive rates.

For further benchmarking, we assessed the performance of our model on data derived from human embryonic stem cells (hESC), for which TSSs and TESs are well annotated in the FANTOM5 database^15^ and PolyA_DB3^16^, respectively. As genomic sequence features can specify the locations of TSSs and TESs, we added 650 and 150 features reflecting functional motifs associated with the choices of TESs and TSSs (see “Methods”). Using these databases, with 70% of the data for training and 30% for testing, we found all the models increased the prediction accuracy for TSSs and TESs, up to 86.8 and 84.2% in the RF model (Fig. 1d). In total, after pooling all 23 cells together and applying the machine learning model, we identified 7777 TSS and 9204 TES peaks, which were significantly enriched at annotated TSS and TES regions, respectively (Fig. 1e). Over 83% of identified TSSs were located within 10 bp of TSSs in FANTOM5, and over 72% of identified TESs were within 10 bp of TESs in PolyA_DB3 (Fig. 1f, g). Of note, functional motifs related to known TSSs/TESs were enriched in 100-bp range around the TSSs/TESs identified in this study, even located more than 1 kb away (Supplementary Tables 3 and 4), suggesting that authentic and unannotated TSSs/TESs in hESCs were identified by scRCAT-seq. Further, we extended the model to other scRCAT-seq datasets from single cells of different sources, such as mouse ESC, mouse oocytes, mouse Dorsal Root Ganglion neurons (DRG), human embryonic kidney 293 cells (HEK293T), and human retinal pigment epithelium (ARPE19) (Supplementary Table 5), and found similar performance in the ability to identify authentic TSSs and TESs (Supplementary Fig. 1e–g), suggesting that our model can be applied to scRCAT-seq datasets of different cell types from different species. Interestingly, the model can also improve annotation of TSS/TES for long-read sequencing datasets, which were derived from mouse oocytes in this study and mouse cerebellum by others (Supplementary Fig. 1e).

To determine the importance of each group of features, we calculated the performance drop after removal of one group at a time from the feature set. This analysis showed that the most important group was related to internal priming, consistent with findings by other studies that internal priming is a major source of false positives^25,27,28^ (Supplementary Fig. 1h). In summary, our results indicate that scRCAT-seq together with machine learning models can identify TSSs and TESs of transcripts with high accuracy, allowing demarcation of transcription boundaries of full-length isoforms.

### The efficiency and sensitivity of scRCAT-seq

We calculated the number of genes detected by scRCAT-seq to assess the efficiency of the method. Compared to existing methods which can detect only a single end of transcripts (5′-TSS or 3′-TES), scRCAT-seq has significantly better or comparable performance in detecting transcripts than methods such as C1 CAGE^19^, STRT-seq^26^ for TSSs (Supplementary Fig. 2a), and BAT-seq^29^ for TES (Supplementary Fig. 2b).

**Fig. 2:**
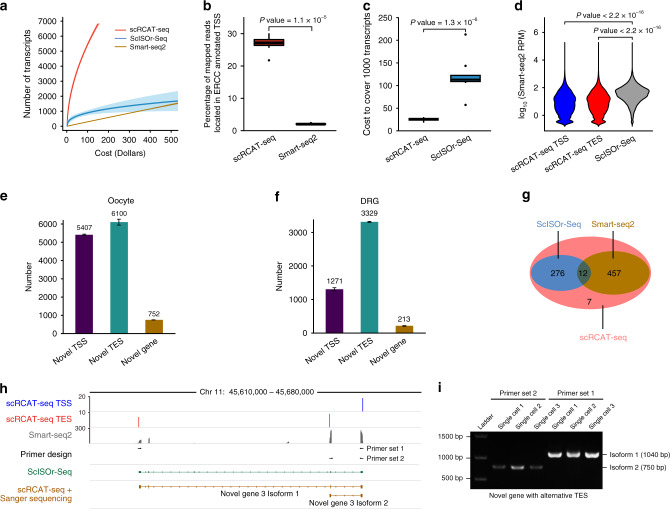
Identification of novel transcripts and isoforms in single cells. **a** The number of transcripts with both ends captured using scRCAT-seq (*n* = 34), Smart-seq2 (*n* = 12), or ScISOr-seq (*n* = 8), versus cost. Shown is the mean number of transcripts shaded by 95% confidence intervals. **b** Comparison between scRCAT-seq (*n* = 10) and Smart-seq2 (*n* = 10) in terms of the ratio of reads covering the 5′ end of transcripts (5-bp range to the end). Significance was computed using two-sided Wilcoxon test. The boxplot shows the median as center line, the interquartile range (IQR) as a box, the whiskers indicate 1.5 × IQR and the outliers as points. **c** The cost of scRCAT-seq (*n* = 18) and ScISOr-seq (*n* = 8) for detection of 1000 transcripts. Significance was computed using two-sided Wilcoxon test. The boxplot shows the median as center line, the interquartile range (IQR) as a box, the whiskers indicate 1.5 × IQR and the outliers as points. **d** Violin plots comparing the expression level between genes detected by scRCAT-seq (*n* = 3) and ScISOr-seq (*n* = 3). Gene expression levels were quantified by Smart-seq2 RPM value. Significance was computed using two-sided Wilcoxon test. **e** Barplot showing the number of novel isoforms of annotated genes and novel, unannotated transcripts in mouse oocytes. The number of transcripts for each category is indicated above the box. Error bars represent standard deviation of the mean (*n* = 3). **f** Barplot showing the number of novel isoforms of annotated genes and novel, unannotated transcripts in mouse DRG. Error bars represent standard deviation of the mean (*n* = 3). **g** Venn diagram for novel transcripts detected concordantly by scRCAT-seq, Smart-seq2, and ScISOr-seq. **h** Genome browser track for an example of a novel gene with alternative polyadenylation sites on a different exon. **i** Gel image showing validation result of novel gene in (**h**). Experiments were repeated three times with similar results. Source data are provided as a Source Data file.

We next compared the performance of scRCAT-seq to that of Smart-seq2 and ScISOr-seq^17,22^ in profiling the full-length transcripts of single cells. Compared to Smart-seq2, scRCAT-seq is more cost-effective at profiling transcript ends due to its higher efficiency in covering transcripts at both ends (Fig. 2a, b, Supplementary Fig. 2c). In addition, we sequenced 6 single oocytes with the PacBio Sequel platform, with 54,000 circular consensus sequencing (CCS) reads per single cell (Supplementary Table 6), which is higher than that reported previously^17,22^. By normalizing the sequencing depth to the cost for both scRCAT-seq and ScISOr-seq, we found that scRCAT-seq had a much higher efficiency in capturing both ends of full-length isoforms than ScISOr-seq. At an equal cost for 4 million PE150 short-reads from Illumina, 7600 transcripts of 3122 genes were detected by scRCAT-seq, while 1100 transcripts of 919 genes were detected by ScISOr-seq (Fig. 2a, Supplementary Fig. 2d). Alternatively, by directly comparing the cost, we found that scRCAT-seq only requires 1/4.8 of the cost required by ScISOr-seq for coverage of 1000 transcripts (Fig. 2c).

In addition, we found that ScISOr-seq mainly detected the top 25% of highly expressed genes detected by scRCAT-seq, and overlap with scRCAT-seq was better for higher expressed genes (Fig. 2d, Supplementary Fig. 2e). Concordantly, scRCAT-seq generated more consistent data, with a twofold higher overlap ratio between single cells than ScISOr-seq (60% versus 30%) (Supplementary Fig. 2f–h). Altogether, these results indicate that scRCAT-seq is a more cost-effective and reliable approach for detecting both start sites and end sites of full-length transcripts at single-cell level.

### Identification of novel transcripts with scRCAT-seq

Leveraging the capacity to demarcate the boundaries of transcripts, we set out to identify novel isoforms, both alternative TSSs/TESs of annotated genes and novel transcripts of unannotated genes. Data derived from mouse oocytes, mouse DRG neurons, hESC and HEK293T were used for benchmarking (Fig. 2e, f, Supplementary Fig. 3a, b). For annotated genes, we identified both alternative TSSs and TESs events, as evidenced by 5407 novel TSSs and 6100 novel TESs in oocytes (Fig. 2e), and 1271 novel TSSs and 3329 novel TESs in DRG neurons (Fig. 2f). In addition, 752 and 213 novel, unannotated transcripts were identified in oocytes and DRG respectively. In total, 62% (469/752) of novel transcripts detected by scRCAT-seq were validated by Smart-seq2, while 38% (288/752) of them were further validated by ScISOr-seq (Fig. 2g), indicating that scRCAT-seq can identify novel transcripts with higher efficiency than ScISOr-seq. Further, Sanger sequencing on individual full-length cDNAs was performed to validate the novel transcripts (Fig. 2h–i, Supplementary Fig. 3c–f), and reveal alternative splicing events within the full-length isoforms. For example, Fig. 2h shows that scRCAT-seq and Sanger sequencing revealed three novel isoforms differing in first exon choices, which were not characterized by Smart-seq2 or ScISOr-seq (Fig. 2h, i). In summary, scRCAT-seq can accurately identify not only novel TSSs and TESs, but also unannotated full-length transcripts in single cells.

### Cell-type-specific transcripts revealed by scRCAT-seq

**Fig. 3:**
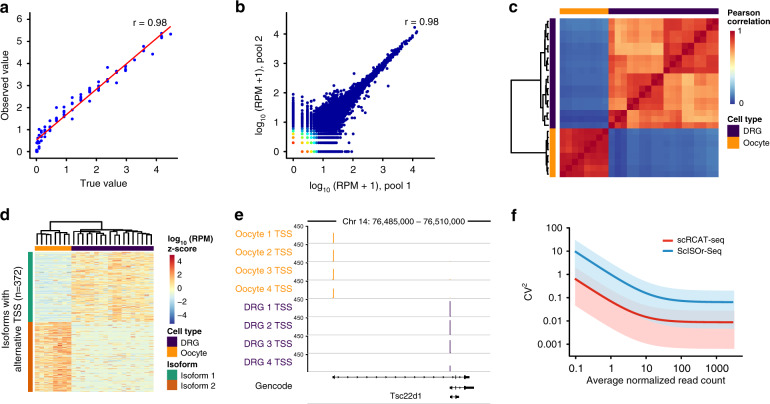
Quantification of RNA isoforms with alternative TSSs and TESs. **a** Scatterplot of observed transcript expression levels (*y*-axis) and true abundance (*x*-axis) of ERCC spike-ins through 5′-end quantification (*n* = 92). Each point represents a transcript. The Pearson’s correlation coefficient is shown in the upper right corner. **b** Scatterplot shows the Pearson’s correlation of transcriptional level of isoforms between replicated pools of three single cells. **c** Heatmap for Pearson’s correlation coefficient of transcriptomes of DRG neuron and oocytes, based on 5′-end quantification of RNA isoforms. **d** Heatmap showing RNA isoforms of alternative TSS choices with cell-type specificity. The major isoforms either in oocytes or in DRG neurons are shown (*n* = 372 isoforms). **e** Genome browser tracks showing the alternative choices of TSS of *Tse22d1* between oocytes and DRG neurons. **f** Squared coefficients of variation of scRCAT-seq (*n* = 4) and ScISOr-seq (*n* = 4), versus the means of normalized read counts. Shown is the mean of squared coefficients of variation shaded by 95% confidence intervals. Source data are provided as a Source data file.

For quantification of isoforms, we count the TSS and TES with either read counts or UMI counts, as the two are highly correlated (Pearson’s correlation coefficient of 0.99) (Supplementary Fig. 4a). As the majority of public protocols for full-length cDNA amplification only label cDNAs with UMI at one end, we first used read counts (normalized as RPM, reads per million) for benchmarking, to count TES and TSS consistently. By comparing the observed value with the known abundances of ERCC mRNA molecules, we found the measured abundances were highly concordant with the ground truth, with a Pearson’s correlation coefficient of 0.98 for both TSS and TES (Fig. 3a, Supplementary Fig. 4b). For the annotated genes of the mouse genome, an internal comparison between random pools of three single cells, each from the oocyte population, gave a correlation coefficient of 0.96 and 0.94 for the quantification of TSS and TES, respectively (Fig. 3b, Supplementary Fig. 4c). Further, we performed cell clustering analysis based on isoform quantification to discriminate different cell types, such as mouse DRG, mouse oocytes, hESC and HEK293T (Fig. 3c, Supplementary Figs. 5a and 6a), and identified isoforms differentially expressed between different cells (Fig. 3d, Supplementary Fig. 5b, c). Comparing DRG and oocytes, we identified 372 isoforms with different TSS and common TES, and 614 isoforms with different TES and common TSS (Fig. 3d, Supplementary Fig. 5b, c). Of note, there is no difference in total expression of the corresponding genes; the difference can only be observed at the isoform level, and isoforms were expressed in a cell-type specific manner (Fig. 3c, Supplementary Fig. 5d–f). In addition to read counts, we also tried to use UMI for quantification, and similar results of isoform choices between HEK293T and hESC were shown when we used either read counts (normalized as RPM) or UMI counts (Supplementary Fig. 6b–e).

Compared to ScISOr-seq, scRCAT-seq has 10-fold lower variance, making isoform quantification much more accurate (Fig. 3f). Of note, as CCS read counts of ScISOr-seq are positively correlated with the number of reads of scRCAT-seq, scRCAT-seq could potentially improve upon performance of ScISOr-seq in accurately quantifying alternative isoforms with lower cost (Supplementary Fig. 4d). For example, with scRCAT-seq data, we can quantify the cell-type specific expression of the isoforms of *Nsf*, which were identified by scRCAT-seq in DRG and oocytes but not by ScISOr-seq due to the limited number of reads (Supplementary Fig. 4e, f).

### Dynamics of isoform choices during human photoreceptor cone development revealed by high-throughput scRCAT-seq

**Fig. 4:**
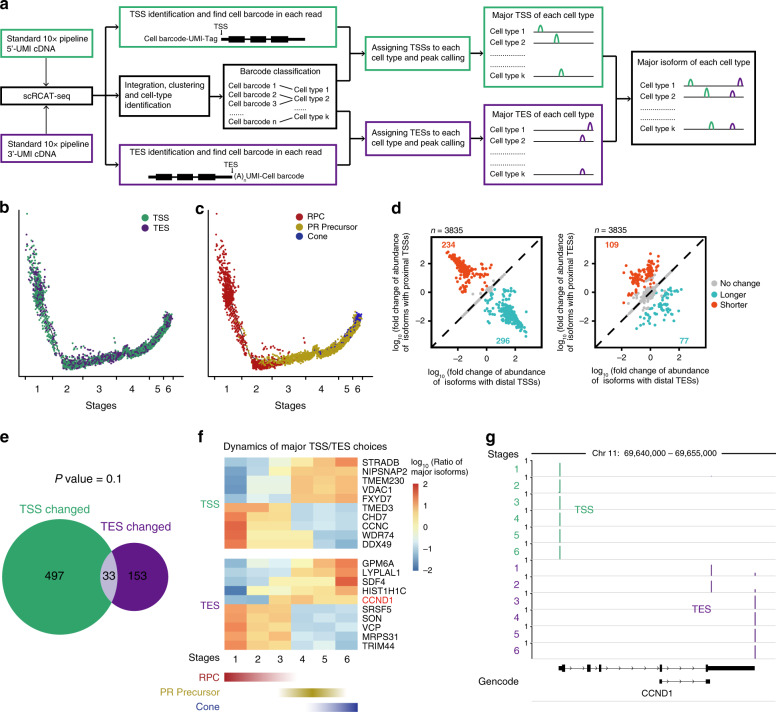
Isoform dynamics during human cone development. **a** Outline of the high-throughput scRCAT-seq. **b** Trajectory plot showing the distribution of TES and TSS data on the trajectory of cone development. Each dot represents a single cell, either from TSS or TES data. **c** Trajectory for the development of cone from RPC generated by using pseudotime analysis with RPC, PR precursor, and photoreceptor cone data. The numbers below show the trajectory divided into stages, to assess the isoform dynamics. **d** Expression data with isoform specificity reveals differential TSS choices (left) and TES choices (right) between cone and RPC. **e** Venn diagram of genes with alternative TSSs and with alternative TESs. Significance was computed using two-sided hypergeometric test. **f** Dynamics of the ratio of major isoforms during the development of cones from RPC. Examples of isoforms with significant differential choices of TSS/TES between RPC and cone are shown, with dynamics for TSS and TES choices in upper and lower panel respectively. The color shows the logNormalized ratio of major isoforms of RPC in each stage. **g** Genome browser track showing the representative gene *CCND1*, where two isoforms differ by a switched TES choice over the time course of cone development. Source data are provided as a Source data file.

We next employed scRCAT-seq to profile a much larger number of single cells by adopting the 10x Genomics droplet platform, which has been widely used for RNA profiling of thousands of single cells in parallel. Single cells within a heterogeneous population were labeled by cell barcodes at one end (5′- or 3′-) of the full-length cDNAs, with 10x Chromium Single Cell 5′ and 3′ kits, respectively. Libraries were generated and sequenced, and data were processed following the scRCAT-seq protocol. Cell subtypes were identified based on 5′- or 3′-transcriptome analysis, and TSSs/TESs of isoforms within each cell were called and assigned to the corresponding cell subtypes. Then, the major TSSs and major TESs were matched to define the major isoforms of each subtype at a population level (Fig. 4a). By doing so, the cost per single cell was reduced to <0.8 dollar per single cell.

For benchmarking, we tested the pipeline with 14,196 single cells, including hESC, HEK293T, ARPE19, mESC for TSS analysis, and human retinal organoids for both TSS and TES analysis. The RF algorithm trained with hESC performs well with an accuracy of around 80% (Supplementary Fig. 7a). The majority of TSS and TES identified were near the annotated TSS and TES (Supplementary Fig. 7b–f). Novel TSSs and TESs within known genes and previously unannotated genes were also identified. For example, we identified thousands of novel TSSs in HEK293T, hESC, APRE, and mESC (Supplementary Fig. 7g). Within 9802 single cells of human retinal organoids, 6628 novel TSSs, 3780 novel TESs, and 259 novel genes were identified (Supplementary Fig. 7h).

In total, 3407 and 6395 single cells were collected for 5′-TSS and 3′-TES analysis, respectively, randomly distributed into six subtypes (Fig. 4b, Supplementary Fig. 8a). Based on the expression pattern of marker genes, the six subtypes were matched to RPC, photoreceptor precursor (PR Precursor), Interneuron precursor (IN precursor), photoreceptor cone, retinal ganglion cells (RGC), and horizontal cell (HC) (Supplementary Fig. 8b, c). We further looked into the differences in TSS/TES choices between different subtypes, and dynamics of isoform switching during cone development from RPC. Pseudotime analysis on RPC, photoreceptor precursor, and photoreceptor cone data confirmed the trajectory from RPC to photoreceptor precursors, and then to cones (Fig. 4b, c, Supplementary Fig. 8d). By comparing RPC and photoreceptor cone, we found 234 genes and 296 genes switched to proximal and distal TSSs respectively, and 109 genes and 77 genes switched to proximal and distal polyadenylation sites respectively (Fig. 4d). TSS switching seems to be more frequent than TES switching, and the two events are not significantly correlated during cone development (Fig. 4e).

To assess the dynamic switching of major isoforms during cone development, we divided the trajectory of cone differentiation from RPC into six stages, with stages 1–3 corresponding to the time course for transition from RPC to PR precursors, and stages 5–6 from PR precursors to cone. The subtypes identified by TSS data and TES data at each stage were highly similar, suggesting TSS data and TES data matched well at similar subtypes (Supplementary Fig. 8e). Major TSSs/TESs of the cells at each stage were determined by binning all the single cells together and calculating the ratio of the major isoform in RPC to that in cones for each gene (Fig. 4e, f). We observed that major isoforms (mainly TSS) switch gradually in the majority of genes, and in most cases, the two isoforms reach equal levels when RPCs turn into photoreceptor precursors (Fig. 4f, Supplementary Fig. 8f, g). A representative example is Cyclin D1 (*CCND1*), which has two isoforms (CCND1a and truncated CCND1a) that differ in TES choice. Its major isoform switches at the later RPC stage before turning into PR precursors: The proximal isoform of CCND1a (truncated CCND1a) is expressed in RPC, and the distal isoform (CCND1a) is expressed in PR precursors and cone (Fig. 4g). Previous studies have shown that truncated CCND1a is the major isoform expressed in cancer cells and promotes cell proliferation and cell-cycle progression^30^. The isoform switch from truncated CCND1a to CCND1a may suggest that *CCND1* mediates differential cell-cycle properties between RPC and PR precursors. Consistent with this hypothesis, our recent study showed that *CCND1* plays a critical role in promoting the G1–S transition of the cell cycle during retinal neurogenesis^31^. In summary, scRCAT-seq can be performed in a high throughput manner to profile thousands of single cells, to identify differential isoform choices among various cell subtypes within a tissue, and to reveal the dynamics of isoform switching during cell fate transitions.

## Discussion

scRCAT-seq, based on short-read sequencing, offers a cost-effective and efficient approach to identify and quantify RNA isoforms in single cells. The accuracy of TES and TSS peaks called by scRCAT-seq is substantially improved when coupled to a machine learning algorithm that filters out the noisy false-positive peaks. Previously, machine learning has been successfully used to predict differential alternative splicing^32,33^, but none of them have been developed for the purpose of identifying authentic demarcations of RNA isoforms to elucidate the transcriptomic complexity of single cells. Furthermore, the model trained in this study also improves the accuracy of other methods to over 90%, as evidenced by the ERCC data from C1 CAGE^19,26^ and C1 STRT^26^, indicating that our model can be applied to other datasets that contain previously unrecognized high false-positive signals. As a result, the accuracy of our approach for quantification of alternative isoforms is very high, as the measured abundances are highly concordant with the ground truth, with a Pearson’s correlation coefficient of 0.98. In summary, scRCAT-seq provides an unprecedented opportunity for detection of previously unannotated genes and unidentified alternative TSSs and TESs, as well as for quantitation of cell-type specific RNA isoforms.

Another clear advantage of scRCAT-seq is its efficiency. Based on short-read sequencing, scRCAT-seq can identify TSSs and TESs simultaneously from sequencing data derived from a single library, enabling investigation of both transcription initiation and polyadenylation in a large number of single cells. Compared with methods which capture only single ends of RNA transcripts, either the TSS or TES, scRCAT-seq is demonstrably better for elucidating transcriptome complexity.

Compared to Smart-seq2, which is mainly used to profile full-length cDNAs of single cells, our approach has much higher efficiency in demarcating the boundaries of transcripts, due to the fact that reads of scRCAT-seq are mainly located at the ends of the transcript while Smart-seq2 reads are mainly located in the middle. Our study also suggests that scRCAT-seq and Smart-seq2 can complement with each other to better illustrate the full-length cDNAs of single cells. Compared to the recently developed long-read sequencing-based method ScISOr-seq, which can profile full-length transcripts for a group of single cells^17,22^, our approach requires 1/4.8 of the cost to detect the same number of transcripts, with higher efficiency. In addition, ScISOr-seq requires at least 1 μg of cDNA input, necessitating extensive amplification of cDNA with unavoidable PCR bias due to the requirement for extra PCR cycles. This results in a decrease in the number of covered transcripts (a few hundred per single cell) and a lower transcript overlap ratio among single cells. In contrast, scRCAT-seq only requires 0.1 ng of cDNA to achieve sufficient coverage of thousands of genes. Most importantly, it is still challenging to use ScISOr-seq to quantify the isoforms differentially expressed between single cells, as accurate quantification requires deep sequencing that is currently too expensive for many labs. In contrast, our method can accurately quantify the transcripts (*r* = 0.98) at an affordable cost for most labs. Due to the high accuracy and efficiency of scRCAT-seq in identifying transcript ends, scRCAT-seq also offers an efficient pipeline for full-length characterization of novel isoforms after targeted construction of full-length cDNA libraries, simply by PCR from the terminal sites identified by scRCAT-seq in single cells. In summary, the performance of scRCAT-seq is a significant improvement upon that of ScISOr-seq in terms of cost, efficiency, and accuracy of both identification and quantification of RNA isoforms.

In this study, we provide two strategies to implement scRCAT-seq. The first is performed on microfluidic platforms to profile thousands of single cells at a time, with a cost of only 0.8 dollar per single cell. Instead of assessing all the isoforms for every single cell, it profiles the major isoforms for subtypes of single cells at population level, and is suitable for characterizing the differential usage of major isoforms between subtypes for a large number of heterogeneous single cells. The second strategy is performed on each single cell separately, with low throughput (hundreds of single cells at a time) and relatively high cost (28 dollars per cell). This strategy is suitable for assessing the differential choices of TSS/TES between individual single cells, especially in studies with limited number of samples, such as oocytes and preimplantation embryos.

Like all technologies, scRCAT-seq has its limitations. First, the initial accuracy of TSS and TES identification is dependent on the effective cloning of full-length cDNA. Although we adapted a widely used method Smart-seq2 to obtain cDNA, other protocols with better performance may be substituted to get full-length cDNA in the future. Second, in some cases, for genes with both multiple TSSs and multiple TESs, it becomes difficult to establish one-to-one matches between the TSS and TES, which may limit the capability to link TSS and TES. A possible solution is to ligate the two ends after cDNA amplification, and construct the libraries with both TSS and TSS and sequence them on the same reads^23^, which we are working on now. Third, scRCAT-seq alone cannot identify the differences in exon splicing, especially for unannotated transcripts. Whereas the information of full-length isoforms of novel genes can be revealed by PCR using primers targeted to transcript ends identified by scRCAT-seq, in this study we multiplexed only a small number of example genes. However, profiling full-length transcripts with higher multiplexing can be done by complementing ScISOr-seq downstream of scRCAT-seq, in order to efficiently profile the targeted amplified full-length cDNA libraries. Including the scRCAT-seq approach to initially identify isoforms of interest will help increase the efficiency of ScISOr-seq, with lower cost.

In conclusion, we believe that this robust and cost-effective approach is an ideal technology for comprehensive and systematic assessment of RNA isoform dynamics across heterogeneous single cells and biological conditions. Not only can it help define cell types with specific isoform expression patterns, but it can also help establish a multi-faceted mammalian cell atlas in conjunction with other methodologies to identify tissue-specific epigenetic elements, genotypes, and cis-elements. Its cost-effectiveness and efficiency allow it to be widely implemented and it may play important roles in projects such as the Human Cell Atlas.

## Methods

### Single-cell isolation

The experiment was performed on 4–6-week-old C57BL/6 mice of both genders. All animal procedures complied with relevant ethical regulations for animal testing and research, were conducted in approval of the Institutional Animal Care and Use Committee (IACUC) of the Zhongshan Ophthalmic Center of Sun Yat-sen University (2018-171). Mice were maintained under standard conditions (12 h light and dark cycles, with sufficient food and water). To obtain single DRG neurons, euthanasia was performed by CO_2_ and cervical dislocation, L4-L5 DRG from mice of both sides were dissected and dissociated into single cells. Single DRG neurons were manually picked by using a micro-capillary pipette. Single cells were incubated into a 0.2-ml thin-wall PCR tube containing 4 μl Smart-seq2 lysis buffer according to the published protocol^21,34^. To obtain postovulatory-aged oocytes, female mice were administered intraperitoneal injections of 10 IU pregnant mare serum gonadotropin and 10 IU human chorionic gonadotropin 48 h later. Cumulus-oocyte-complexes (COCs) were collected 24 h after human chorionic gonadotropin injections from the oviductal ampullae. All cumulus cells were removed from the oocytes enzymatically by trypsin treatment (Sigma-Aldrich) for 2 min and oocytes were subsequently washed in DMEM medium containing 10% fetal bovine serum (FBS) (Sigma-Aldrich). Oocytes were picked into a 0.2-ml thin-wall PCR tube contains 4 μl Smart-seq2 lysis buffer as described before.

### scRCAT-seq library construction for a single cell

The full-length cDNA was generated through reverse transcription with transcriptase III and the RT primer (5′-AAGCAGTGGTATCAACGCAGAGTN8[16 bps of cell barcode]T30VN-3′), followed by PCR amplification according to the Smart-seq2 protocol^21^, with the minor modification that Superscript II was replaced by Superscript III to improve the yield of cDNA. ERCC RNA spike-in Mix which contains 92 transcripts (Thermo Fisher) was added and processed in parallel with poly-A RNA. After purification, 0.1 ng cDNA was used for tagmentation with the Nextera XT DNA sample preparation kit (Illumina) and fragments of both ends of the cDNA were selectively amplified by using the P5 index primer (5′AATGATACGGCGACCACCGAGATCTACAC[8 bps of index]TCGTCGGCAGCGTCAGATGTGTATAAGAGACAGGTGGTATCAACGCAGAGT) and the P7 index primer (5′CAAGCAGAAGACGGCATACGAGAT[8 bps of index]GTCTCGTGGGCTCGG) as shown in Fig. 1a. Library are purified using 1.8× Agencourt AMPure XP beads (BECKMAN COULTER), and then loaded on an E-Gel 2% SizeSelect, and fragments of a length of 200–300 bp bases were selected. Simultaneously, 0.1 ng of cDNA was used to generate standard Smart-seq2 libraries and sequencing for validation. Library was assessed by using Agilent Bioanalyzer 2100, and sequenced on Illumina Xten platform in PE150 model. The rest of the cDNA of mouse oocytes and DRG neurons were used for PacBio ISO-seq for comparison in parallel.

### Single-cell ISOr-seq

Single-cell ISO-seq was performed on PacBio Sequel platform. Full-length cDNA of eight single cells were mixed together to reach the total amount of 2 μg for each flowcell. PacBio library construction is done by using SMRTbell Template Prep Kit (PacBio cat#100-991-900), and sequenced using SMRTcells (PacBio cat#101-008-000), with eight single samples per SMRTcell.

### Culture of cell lines

E14Tg2a mESC line was maintained in 2i medium, consisting of DMEM supplemented with 15% FBS, 0.1 mM β-Mercaptoethanol (Sigma), 1000 U/ml LIF (Millipore), 1 μM PD0325901, and 3 μM CHIR99021 (both from Selleckchem). The feeder-free E14Tg2a mESC line was cultured on 0.1% gelatin. 0.05% Trypsin/EDTA was used to passage the cells at the confluency of 80%. Human ESC line H9 was kindly provided by Stem Cell Bank, Chinese Academy of Sciences. Undifferentiated hESCs were cultured in Essential-8 (E8) medium (Invitrogen) on Vitronectin (VTN-N)-coated 6-well plates. When reaching over 80% confluency, cells were passaged using Versene (Invitrogen) and split normally twice a week. ARPE19 and HEK293T were cultured in a medium consisting of DMEM supplemented with 5% FBS, nonessential amino acids, and penicillin-streptomycin. 0.05% Trypsin/EDTA was used to passage the cells at the confluency of 80%.

### HESC-derived retinal organoid differentiation

To initiate retinal differentiation from hESC, colonies were dissociated into small cell clusters with dispase (2 mg/ml), and allowed to reaggregate in a medium which was gradually switched from E8 to neural induction medium (NIM: DMEM/F12 [1:1], 1% N2 supplement, MEM nonessential amino acids, penicillin-streptomycin, and 2 mg/ml heparin sulfate) over 4 days. On day 6, recombinant human BMP4 (50 ng/ml) was added into NIM medium to increase the efficiency of retinal differentiation, which was diluted by a half-medium change every third day. Cell aggregates were attached to 6-wells plates on day 7 with medium containing 10% FBS. On day 16, neural rosettes were dislodged from plates with 10 μl tip manually and henceforth maintained in retinal differentiation medium (RDM: DMEM/F12 [3:1], 2% B27 supplement, MEM nonessential amino acids, and penicillin-streptomycin) to allow the formation of retinal organoids. From day 30, culture medium was supplemented with 10% FBS, 100 mM taurine, 2 mM GlutaMAX, and 0.5 mM retinoic acid for long-term retinal organoid culture.

### Cell dissociation for 10x

Retinal organoids were dissociated using Accutase at 37 °C for 30 min, while hESC and mESC were dissociated using Accutase at 37 °C for 5 min to acquire a single-cell suspension. After being strained through the cell strainer, collected cells were resuspended in PBS containing 0.04% bovine serum albumin. scRNA-seq libraries were prepared following manufacturer’s instructions (single-cell gene expression 3′ V3 or 5′ kit of 10x Genomics). In brief, single cells were partitioned into GEM followed by cell lysis, reverse transcription of RNA, cDNA amplification, and library construction steps. Libraries were sequenced on Illumina HiSeq 2500 platforms.

### Data processing of next-generation sequencing data

TSS and TES raw data were extracted and processed separately. For TSS data, reads with the sequencing tag 5′-GTGGTATCAACGCAGAGTACATGGG-3′ were selected, and TSO sequences 5′-GTGGTATCAACGCAGAGTACAT-3′ were trimmed away. Then, these reads were aligned to human genome (hg38) or mouse genome (mm10) with STAR^35^ (version 2.7.3a) with parameters (--outFilterMultimapNmax 1 --outFilterScoreMinOverLread 0.6 --outFilterMatchNminOverLread 0.6). Uniquely mapped reads were kept. Reads that aligned to the ribosomal RNA region were also discarded.

For the TES data, we first processed to remove 3′ adaptor sequences with cutadapt^36^ (version 1.18), and then extracted paired reads with R1 having a 3′ Tag and R2 having at least 10 poly-A sequences at the 3′ side. Poly-A sequences at the end of R2 were further trimmed. By using STAR with parameters described above, reads were aligned to human genome (hg38) or mouse genome (mm10). Reads mapped to multiple sites, with low-quality alignment, and aligned to mitochondrial or ribosomal RNA region were discarded.

For Smart-seq2 data, raw reads past quality control were aligned by STAR using the parameters as described above. Only reads that uniquely mapped to hg38 or mm10 were retained and read count on each gene in each sample was computed using HTSeq^37^ (version 0.11.2). Differentially expressed gene analysis was performed using SCDE^38^ (version 2.10.1).

For comparison, we downloaded BAT-seq data, C1 STRT data, and C1 CAGE data. For the BAT-seq data, we picked 192 mouse ES cells. For the C1 STRT data, 80 mouse brain cells from the single-cell dataset were randomly picked. For the C1 CAGE data, we picked 92 mouse ES cells. Same strategies were used with small modification to process BAT-seq, C1 STRT data, and C1 CAGE data. For all data, we converted bam files to bed files with BEDtools^39^ (version 2.27.1). For 5′ end data, we extract the 5′ end from bed files for further analysis. Likewise, we extract the 3′ end from bed files for 3′ end data.

### Data processing of ScISOr-seq data

Circular consensus reads (CCS) were obtained from the raw data of subreads Bam files by using PacBio Sequel SMRT-Link 7.0 Soft, with the default setting of parameters: minLength 10, maxLength 21000, minReadScore 0.75, minPasses 3. Then, reads were considered Full-length, non-concatemer (FLNC) if they contained 5′ and 3′ primers in addition to a poly-A tail. Primer and poly-A tails were removed by cutadapt^36^. Further, FLNC reads were aligned to reference genome mm10 using Minimap2^40^ (version 2.17) with parameters (-t 30 -ax splice -uf --secondary=no -C5 -O6,24 -B4). CCS count on each gene in each sample was computed using HTseq. The output Sam files were fed into Cupcake ToFU to collapse the mapped FLNC reads into unique transcripts. Scripts are available at https://github.com/Magdoll/cDNA_Cupcake. Eventually, isoforms were identified and filtered using SQANTI2 (version 7.4.0) against mm10 transcriptome annotation.

### Peak calling

To identify TSSs and TESs, we used the CAGEr (version 1.24.0) package in R. Peaks were called using distclu (threshold = 3, nrPassThreshold = 1, thresholdIsTpm = TRUE, removeSingletons = FALSE, keepSingletonsAbove = 10, maxDist = 20). The position of dominant TSS/TES in each peak was set to represent the position of peak. TSS and TES annotation reference was based on gencode release_M18, and peaks mapped between 2 kb upstream the annotated TSSs and 2k downsteam the annotated TESs were considered to belong to the said gene. We then extracted 5′-end and 3′-end of all annotated transcripts and converted to bed files with a custom R script, and the distance between the called peaks and the nearest annotated TSS/TES was calculated by a custom script.

### Machine learning analysis

To predict peaks as real or false TSSs/TESs, we employed four widely used supervised classification models: LR, RF, KNN, and SVM models^41–43^.

### Input and data preprocessing

With the peaks as the input, we generated three major types of features, which were related to the characteristics of the peaks. First, features related to read distributions along the whole transcript, such as the peak abundance, peak width, height of the peak etc. were generated. We applied necessary normalization steps including minmax, and quantile normalization to these raw features to make them in the range of [0, 1] before feeding them to the machine learning models. Second, features related to the appearance of strand-specific motifs related to authentic TSSs/TESs were included. For TSS peaks, we searched for BREu (SSRCGCC), TATA-box (TATAWAWR), and BREd (RTDKKKK) motifs upstream, allowing up to 2 mismatches. Genomic sequences located up to 50 nt upstream were extracted, and parsed by a custom python script to calculate the frequency and location of each motif, and 150 (3 × 50) features were generated. For TES, we searched for 2 canonical polyadenylation signals (AATAAA and ATTAAA) and 11 non-canonical polyadenylation signals (AAGAAA, AATAGA, AATACA, AATATA, AATGAA, AGTAAA, ACTAAA, GATAAA, CATAAA, TATAAA, and TTTAAA), and generated 650 (13 × 50) features within 50 nt sequences around the peaks. Third, features related to false-positive peaks such as the internal priming sites during reverse transcription, and internal sites for template switching, were generated with a customized python script.

Last, we assigned the label “TRUE” for peaks corresponding to authentic TSSs/TESs annotated in FANTOM5 database^15^ and PolyA_DB3^16^, and the label “FALSE” for peaks that were not annotated. The machine learning models were trained on these labels with the features described above.

### Training models

The data were randomly split into a training set (70%) and a test set (30%). The test set was used to evaluate the model fit. We utilize the popular open-source python machine learning library scikit-learn to train the models. A fivefold cross-validation was conducted on the training set to select hyperparameters. Specifically, we tried to find the best hyperparameter sets for each machine learning algorithm in TES/TSS data through two rounds of GridSearchCV. In the first round, we used coarse-grained search to find the best range of hyperparameter sets, and then use fine-grained search to find the best hyperparameter set based on the previously found range. After that, the best hyperparameter sets are used to train the machine learning algorithms using the whole training set data. All models’ performance was evaluated with accuracy, and we selected the model with the best performance.

### Assessing performance of the models

Once the models were properly trained, we used them to predict the real data, including the TSSs and TESs. The performance was estimated by Accuracy (Acc).

$${\mathrm{Acc}} = ({\mathrm{TP}} + {\mathrm{TN}})/({\mathrm{TP}} + {\mathrm{TN}} + {\mathrm{FP}} + {\mathrm{FN}}),$$

where TP is true positives, TN is true negatives, FP is false positives, and FN is false negatives.

In order to determine the ability for the model to be generalized across datasets derived from various cell types, we obtained a trained model using hESC data, and measured its performance on several other datasets, such as TES/TSS data from human HEK293T and ARPE, mouse DRG and oocyte, and mESC.

### Quantification of cell-type-specific isoforms

Expression values for each peak (TSS/TES) were quantified as reads per million (RPM) generated by CAGEr. To identify cell-type-specific isoforms, the major TSS/TES positions of genes co-expressed between the two types of cells are compared by intersecting the bed files of each with BEDtools^39^. Genes with either alternative TSS or alternative TES between the two were selected. Then, the differential expression analysis on the RPM value of the major isoform of each cell type between the two was performed with DESeq2^44^ (version 1.26.0).

### Sequencing full-length cDNA of target genes

Primers were designed according to the coordinates of TSS/TES identified by scRCAT-seq. Full-length cDNA of all isoforms of a target gene was amplified by PCR from the cDNA pool of single cells generated with Smart-seq2. Briefly, 1 ng full-length cDNA was used to perform 35-cycle PCR with Premix Taq^TM^ (TaKaRa). PCR products were purified with QIAquick Gel Extraction Kit (Qiagen) and Sanger-sequenced with corresponding primers. All assays were performed for three individual single-cell samples. PCR primers used for novel genes are listed in Supplementary Table 7. The original uncropped gel images are presented in Supplementary Fig. 9.

### Data processing of droplet-based single-cell RNA sequencing data

The 10x droplet sequencing data were processed using the Cell Ranger (version 3.1.0) pipeline from 10x Genomics. In brief, reads were demultiplexed and aligned to the GRCh38 or mm10 genome. UMI counts were quantified to generate a gene-barcode matrix. Cells were filtered to remove those containing less than 200 genes. Genes that were detected in less than 3 cells were also removed. Further analyses of these cells were performed using the Seurat^45^ (version 3.1.0) R packages, as described in the tutorials (https://satijalab.org/seurat/). Briefly, cells were normalized using LogNormalize and multiplied by a scale factor of 10,000. HVGs (high variable genes) were identified and used for further analysis. Shared cell states were identified using integration procedure in Seurat.

Dimensionality reduction was performed using principal component analysis (PCA). Statistically significant PCs were identified using the Jackstraw function. The score of cells in those significant PCs was used to build a k-nearest neighbor (KNN) graph. The Louvain algorithm was used for identifying cell clusters in KNN graph. Uniform manifold approximation and projection (UMAP) dimensionality reduction was used to project these populations in two dimensions. Pseudotime analyses of organoids were performed using the Monocle2^46^ (version 2.12.0) R package. Differentially expressed genes among RPC, PR precursor, and Cone were identified using FindAllMarkers function and used as input for temporal ordering of those cells along the differentiation trajectory.

Dynamics of the isoform choices were evaluated by a trajectory-based calculation of the ratios of the major isoforms, which were mainly expressed either in RPC or in Cone. First, the pseudotime trajectory was divided into six stages, which correspond to the continuous transition from RPC (stages 1–3) to PR precursor (stages 3–6), and then to cone (stages 5–6). Second, the bam file for all the single cells was first converted to a bed file, the cell barcodes and UMI for the single cells were added into the bed file as two columns. Further, the bed file was split into six small subfiles corresponding to six stages by using the cell barcodes of the single cells. The major TSS and major TES of each gene were assessed and matched to identify the major isoform for each cell type. Third, differential choices of major isoforms between RPC and cone were identified. The dynamic transition from major isoform of RPC to major isoform of cone was assessed by calculating the ratio of the two isoforms at the six stages, where isoforms were quantified by counting the end with alternative choices.

### Reporting summary

Further information on research design is available in the Nature Research Reporting Summary linked to this article.

## Supplementary information

Supplementary Information

Reporting Summary

Description of Additional Supplementary Files

Supplementary Software 1

## Data Availability

All sequence data generated in this study are available at Gene Expression Omnibus (GEO) with the accession number GSE134311. Published data from BAT-seq^29^, C1 STRT^47^, and C1 CAGE^19^ were downloaded from GEO (BAT-seq accession number: GSE60768; C1 STRT accession number: GSE60361) or DDBJ database (C1 CAGE accession number: PRJDB5282). FANTOM5 BAM files were downloaded from https://fantom.gsc.riken.jp/5/datafiles/reprocessed/. In total we downloaded seven samples: ARPE19, HEK293T, hESC, adult retina, mESC, mouse dorsal spinal cord, and ovary. PolyA_DB3 annotations were downloaded from https://exon.apps.wistar.org/PolyA_DB/v3/misc/download.php. The data supporting the findings of this study are available from the corresponding authors upon reasonable request. Source data are provided with this paper.

## Source data

Source Data

## Code availability

All custom computer code used in this study is freely available at https://github.com/huyoujinlab/scRCAT-seq. The workflow for processing scRCAT-seq data is provided as a Supplementary Software file.
