# Supplementary material for: Single-cell RNA cap and tail sequencing (scRCAT-seq) reveals subtype-specific isoforms differing in transcript demarcation
Source: Nat Commun. 2020 Oct 13;11:5148. doi: 10.1038/s41467-020-18976-7 (PMC7555861; doi:10.1038/s41467-020-18976-7)
Supplement: Supplementary file 1 — Supplementary Information [file 41467_2020_18976_MOESM1_ESM.pdf]

Supplementary Information for

Single-cell RNA cap and tail sequencing (scRCAT-seq) reveals subtype-specific isoforms differing in transcript demarcation

Youjin Hu[†,*], Jiawei Zhong[†], Yuhua Xiao, Zheng Xing, Katherine Sheu, Shuxin Fan, Qin An, Yuanhui Qiu, Yingfeng Zheng, Xialin Liu, Guoping Fan, Yizhi Liu[*]

[†] These authors contributed equally to this work.

[*] These authors jointly supervised this work.

Correspondence should be addressed to Y.L. (email: yzliu62@yahoo.com) or to Y.H. (email: huyoujin@gzzoc.com).

This PDF file includes:

Supplementary Fig. 1 to 9

Supplementary Table 1 to 7

Supplementary Figures

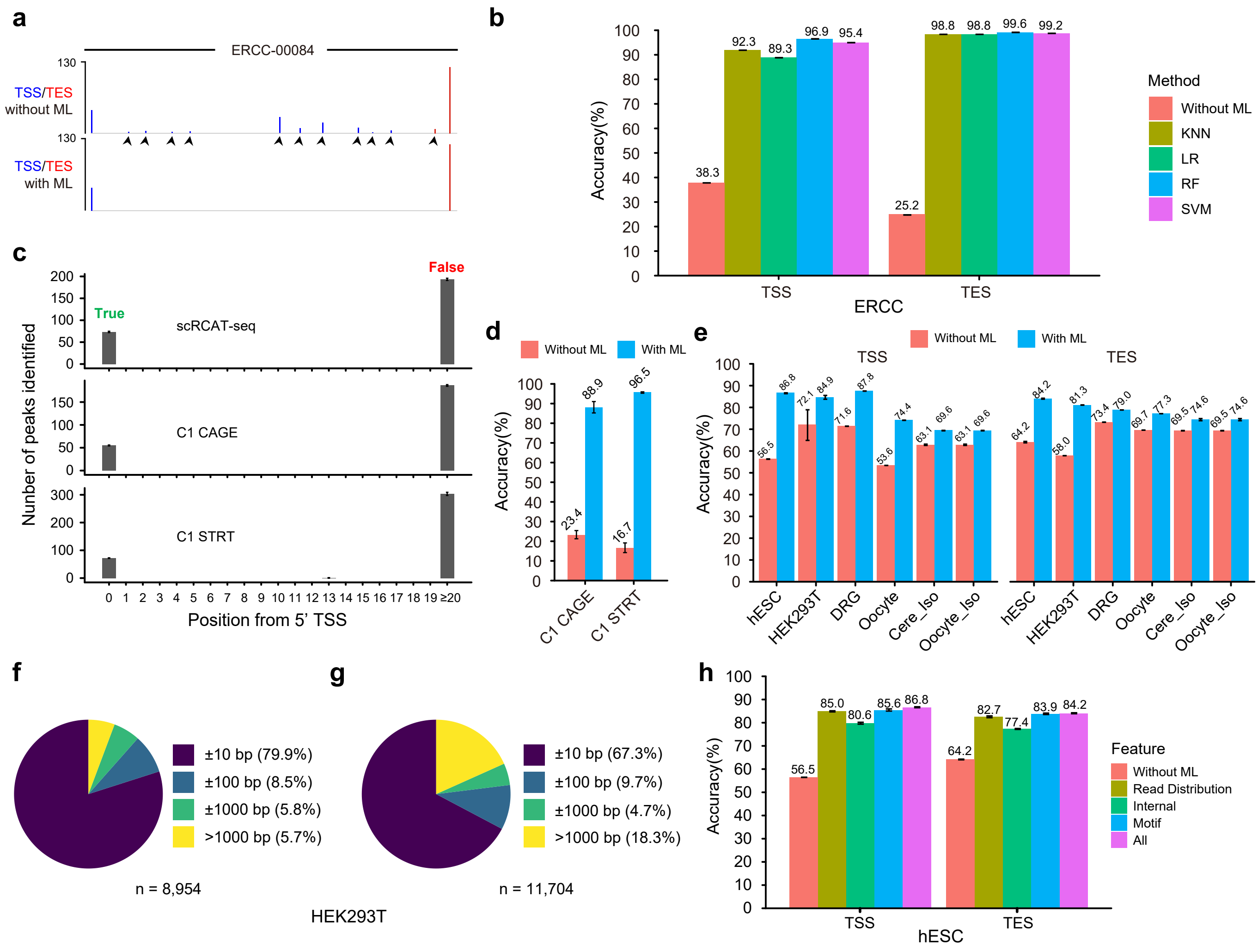

Supplementary Fig. 1. Accuracy to identify TSSs/TESs was improved by ML. a Genome browser track showing a representative example of peaks on ERCC gene, with the false positive peaks filtered out by ML indicated by arrows. **b** Bar plot showing variable performance of different ML algorithms, assessed by accuracy, to predict the TSS and TES of ERCC. Error bars represent standard deviation of the mean (n = 3). **c** Barplot showing TSS peaks identified in the data of scRCAT-seq, as well as public data sets of ERCC for C1 CAGE, C1 STRT. True positive peaks located around the annotated TSSs and false positive TSS peaks located elsewhere are indicated. Error bars represent standard deviation of the mean (n = 3). **d** Barplot showing the improved accuracy in identifying TSSs by the RF model in public ERCC datasets generated by C1 CAGE, C1 STRT. Error bars represent standard deviation of the mean (n = 3). **e** Barplot showing the performance of RF model trained with hESC data on other dataset derived from

mouse DRG, mouse oocytes, HEK293T cells. Cere_Iso represent of ScISOr-Seq data derived from mouse cerebellum (Gupta et al, 2018)[1] and Oocyte_Iso is ScISOr-Seq data in this study. Error bars represent standard deviation of the mean (n = 3). **f** Pie chart illustrating the distribution of the identified TSSs in HEK293T cells relative to the TSSs in the FANTOM5 database. The total number of TSS peaks identified after optimization by the machine learning models is indicated under the pie chart. **g** Pie chart illustrating the distribution of the identified TSSs in HEK293T relative to the TSSs in the FANTOM5 database. **h** Feature importance of different features determined by observing the drop in performance when deleting the corresponding features. Error bars represent standard deviation of the mean (n = 3). Source data are provided as a Source Data file.

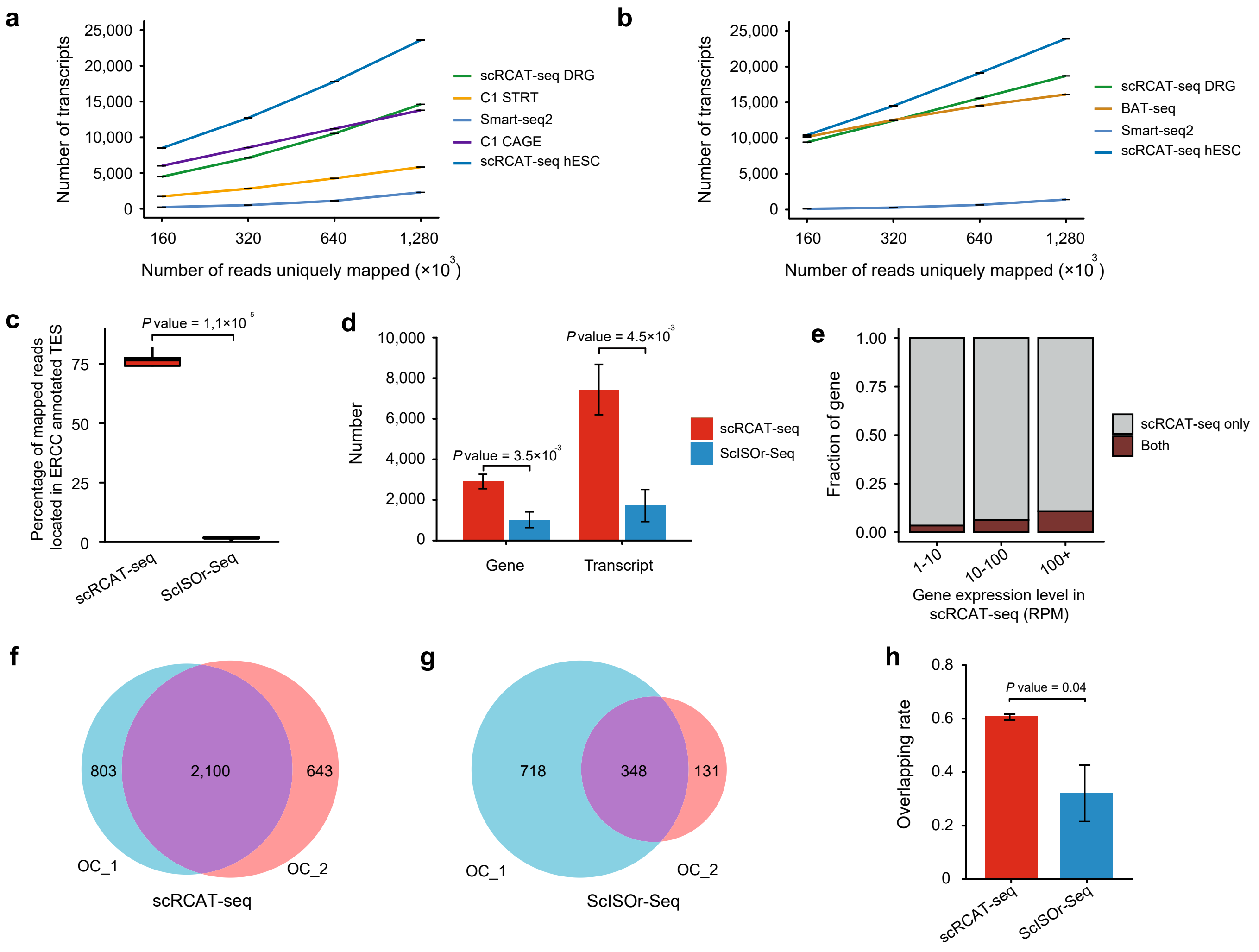

Supplementary Fig. 2. Efficiency and consistency of scRCAT-seq. a The number of transcripts with 5' head detected by scRCAT-seq, C1 STRT, Smart-seq2, and C1 CAGE at variable sequencing depth (n = 10). Error bars represent standard deviation of the mean. **b** The number of transcripts with 3' tail detected by scRCAT-seq, BAT-seq, and Smart-seq2 at variable sequencing depth (n = 10). Error bars represent standard deviation of the mean. **c**, Comparison of scRCAT-seq (n = 10) and Smart-seq2 (n = 10) in terms of the ratio of reads covering 3' end of transcripts within 5 bps. Significance was computed using two-sided Wilcoxon test. The boxplot shows the median as center line, the interquartile range (IQR) as a box, the whiskers indicate 1.5 × IQR and the outliers as points. **d** Number of genes and transcripts covered by scRCAT-seq (n = 3) and ScISOr-Seq (n = 3) respectively. The number of reads for scRCAT-seq was 4 million per single cell and the CCS number for ScISOr-Seq is 50,000 per cell. Significance was computed using two sided t-test. Error bars represent standard deviation of the mean. **e** Stacked barplots showing the number of genes with different expression levels detected in oocytes by scRCAT-seq and ScISOr-Seq (n = 3). **f** Venn diagram for genes detected concordantly among single cells

by scRCAT-seq. **g** Venn diagram for genes detected concordantly among single cells by ScISOr-Seq. **h** Barplot shows the overlap rate of genes detected among single cells, by scRCAT-seq (n = 3) versus ScISOr-Seq (n = 3). Significance was computed using two sided t-test. Error bars represent standard deviation of the mean. Source data are provided as a Source Data file.

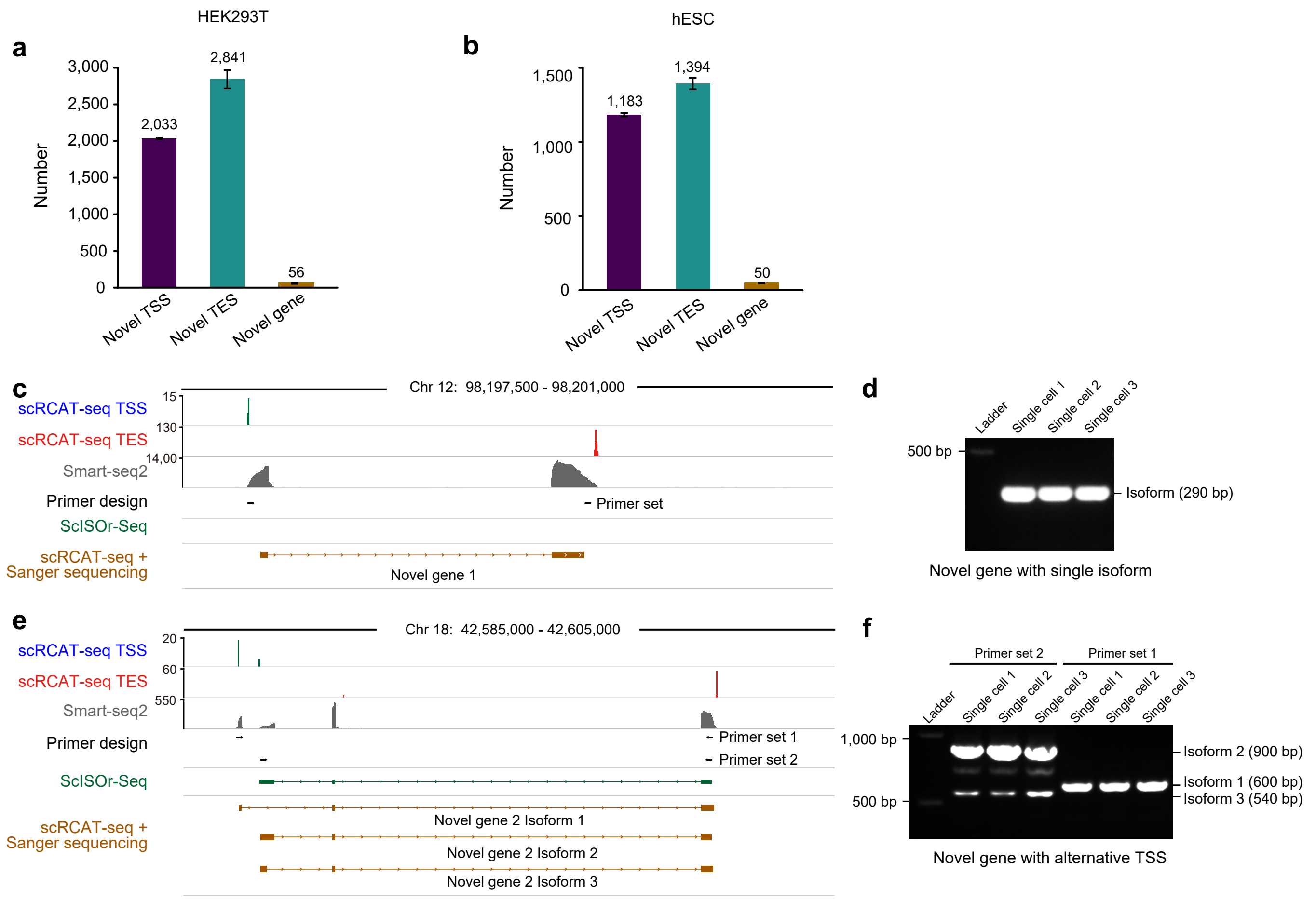

Supplementary Fig. 3. Novel isoforms and genes identified. **a**, **b** Barplot showing the number of novel isoforms of annotated genes and novel, unannotated transcripts in HEK293T cells and hESC. Error bars represent standard deviation of the mean (n = 3). **c** Genome browser track for an example of a novel gene with a single isoform. **d** Gel image showing validation result of the novel gene in **c**. Experiments were repeated three times with similar results. **e** Genome browser track for an example of novel genes with alternative TSSs on a different exon. **f** Gel image showing validation result of the novel gene in **e**. Experiments were repeated three times with similar results. Source data are provided as a Source Data file.

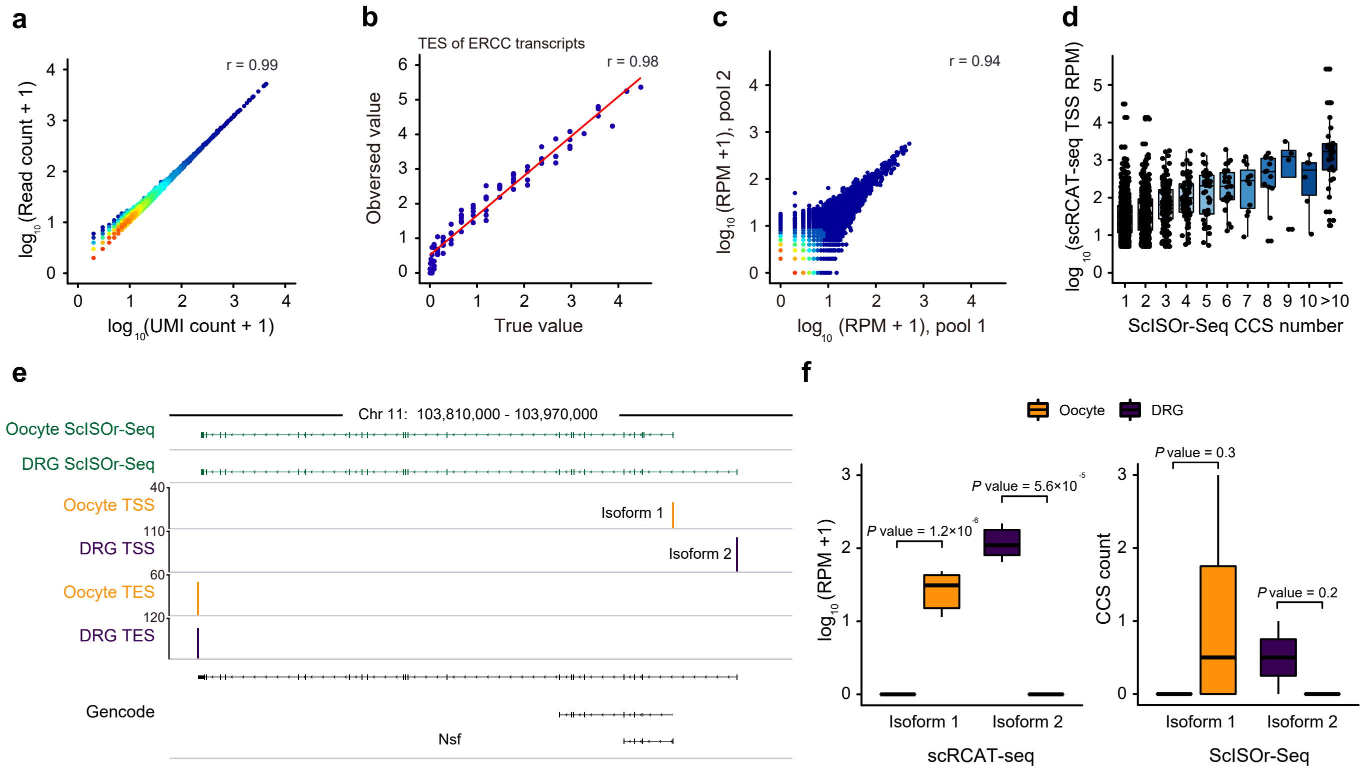

Supplementary Fig. 4. Quantification of RNA isoforms. a Scatter plot of observed transcript expression levels of transcripts in hESC, calculated with read count (y axis) and UMI (x axis). Each dot represent a transcript. The Pearson's correlation coefficient is shown in the upper right corner. **b** Scatter plot of observed transcript expression levels (y axis) and true abundance (x axis) of ERCC spike-ins through 3'-end quantification (n = 92). **c**, Scatter plot shows the Pearson's correlation of transcriptional level of isoforms between replicated pools of 3 single cells. **d** Boxplot for the expression level comparison among genes with different CCS numbers detected by ScISOr-Seq (n = 2). The boxplot shows the median as center line, the interquartile range (IQR) as a box, the whiskers indicate 1.5 × IQR. Individual data points are overlaid on boxplot. **e** Genome browser track showing an example of *Nsf*. which has two isoforms detected by both scRCAT-seq and ScISOr-Seq. **f** Boxplot for the example gene *Nsf,* which has two isoforms differentially expressed in oocytes (n = 8 and 6 for scRCAT-seq and ScISOr-Seq, respectively) or in DRG neurons (n = 18 and 2 for scRCAT-seq and ScISOr-Seq, respectively), while the expression value assessed by ScISOr-Seq is not differential between the two cell types. Significance was computed using two-sided Wilcoxon test. The boxplot shows the median as center line, the interquartile range (IQR) as a box, the whiskers indicate 1.5 × IQR and the outliers as points. Source data are provided as a Source Data file.

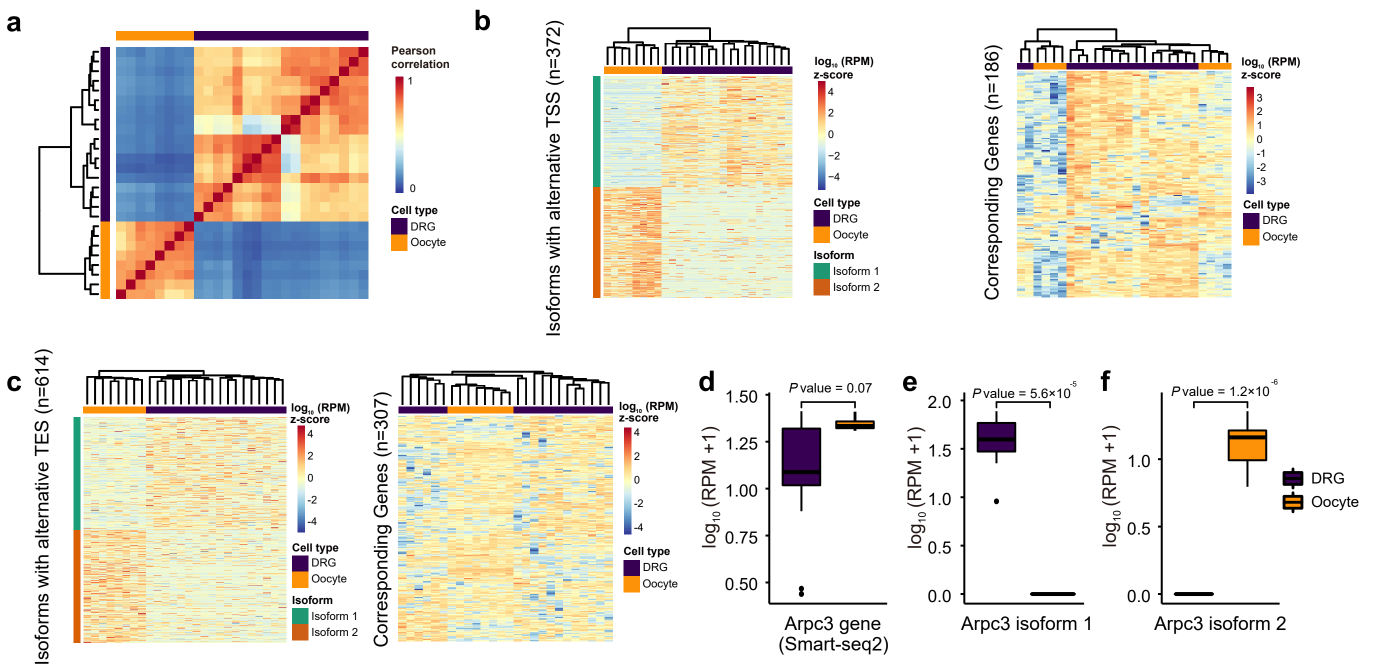

Supplementary Fig. 5. Identification and quantification of cell-type specific isoforms. a Heatmap for Pearson's correlation coefficient of transcriptomes of DRG neuron and oocytes, based on 3'-end quantification of RNA isoforms. **b** Heatmap showing RNA isoforms of alternative TSS choices with cell type specificity (left panel), and the expression of corresponding genes assessed by Smart-seq2 (right panel). **c**, Heatmap showing RNA isoforms of alternative TES choices with cell type specificity (left panel), and the expression of corresponding genes assessed by Smart-seq2 (right panel). **d**-**f** Boxplot for the example gene *Arpc3*, which has two isoforms differentially expressed in oocytes (n = 8) or in DRG neurons (n = 18) (**e**, **f**), while the overall gene expression assessed by Smart-seq2 is not differential between the two cell types (n = 19 DRG neurons and 4 oocytes) (**d**). Significance was computed using two-sided Wilcoxon test. The boxplot shows the median as center line, the interquartile range (IQR) as a box, the whiskers indicate 1.5 × IQR and the outliers as points. Source data are provided as a Source Data file.

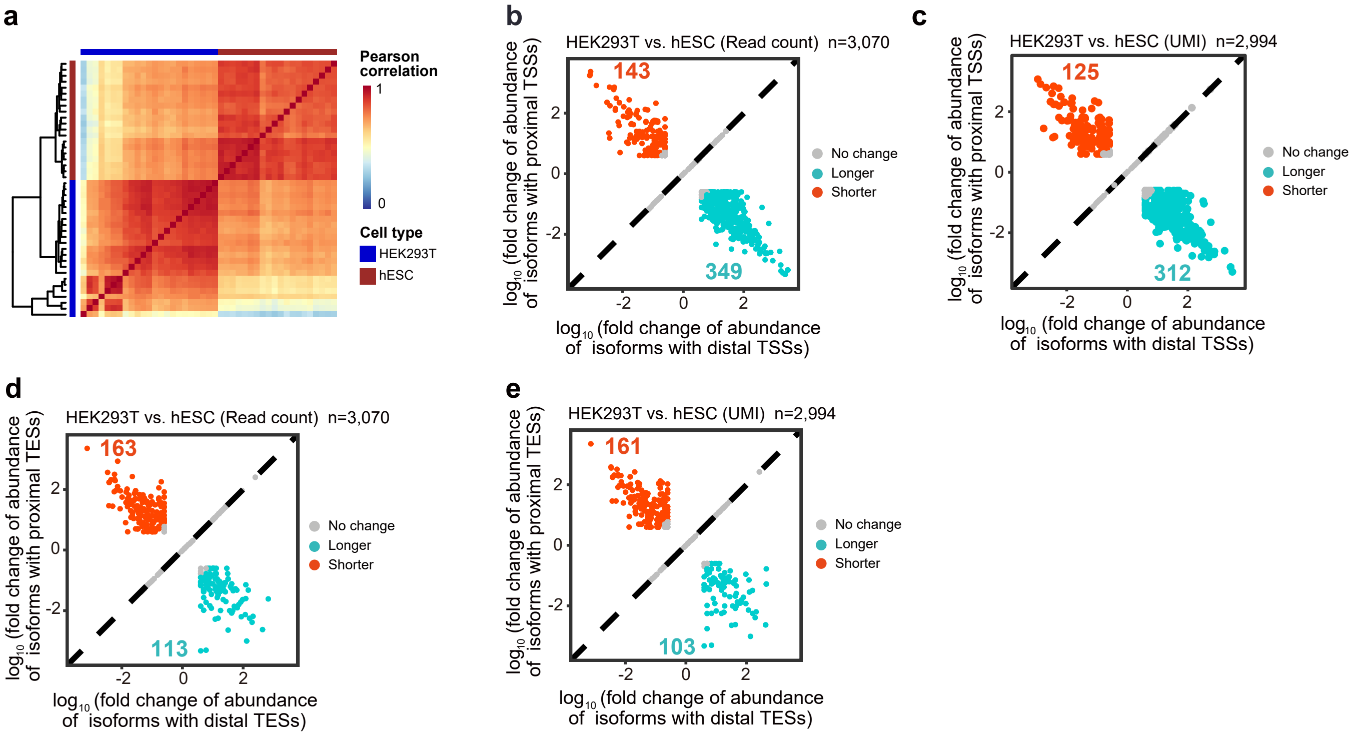

Supplementary Fig. 6. Cell-specific isoforms identified with UMI and Read counting. a Heatmap of correlation between single cell of hESC and HEK293T cells. **b**, **c** Isoforms with alternative TSS in HEK293T versus hESC, based on Read counting and UMI counting respectively. **d**, **e** Isoforms with alternative TES in HEK293T versus hESC, based on Read counting and UMI counting respectively. Source data are provided as a Source Data file.

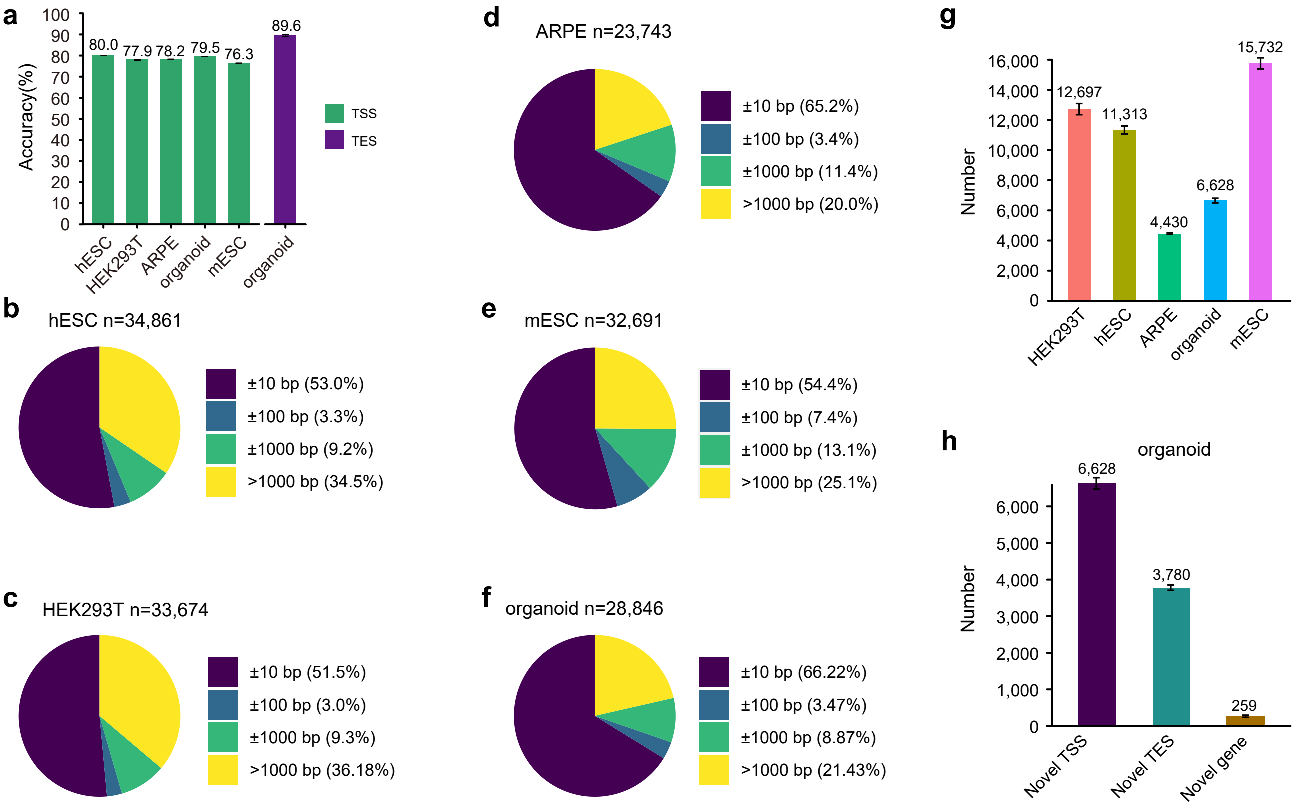

Supplementary Fig. 7. High throughput scRCAT-seq. a Barplot shows the accuracy of high throughput scRCAT-seq. The ML model was trained on hESC and applied to dataset derived from various cells. Error bars represent standard deviation of the mean (n = 3). **b-f** Pie chart of distribution of the identified TSSs from human retina organoids, HEK293T, hESC, ARPE19 and mESC, relative to TSSs annotated in the FANTOM5 database. **g** Barplot showing the number of novel isoforms with unannotated TSSs in HEK293T, hESC, APRE, human retinal organoid and mESC. Error bars represent standard deviation of the mean (n = 3). **h** Barplot showing the number of novel isoforms of annotated genes and novel, unannotated transcripts in human retinal organoid. The number of transcripts for each category is indicated above the box. Error bars represent standard deviation of the mean (n = 3). Source data are provided as a Source Data file.

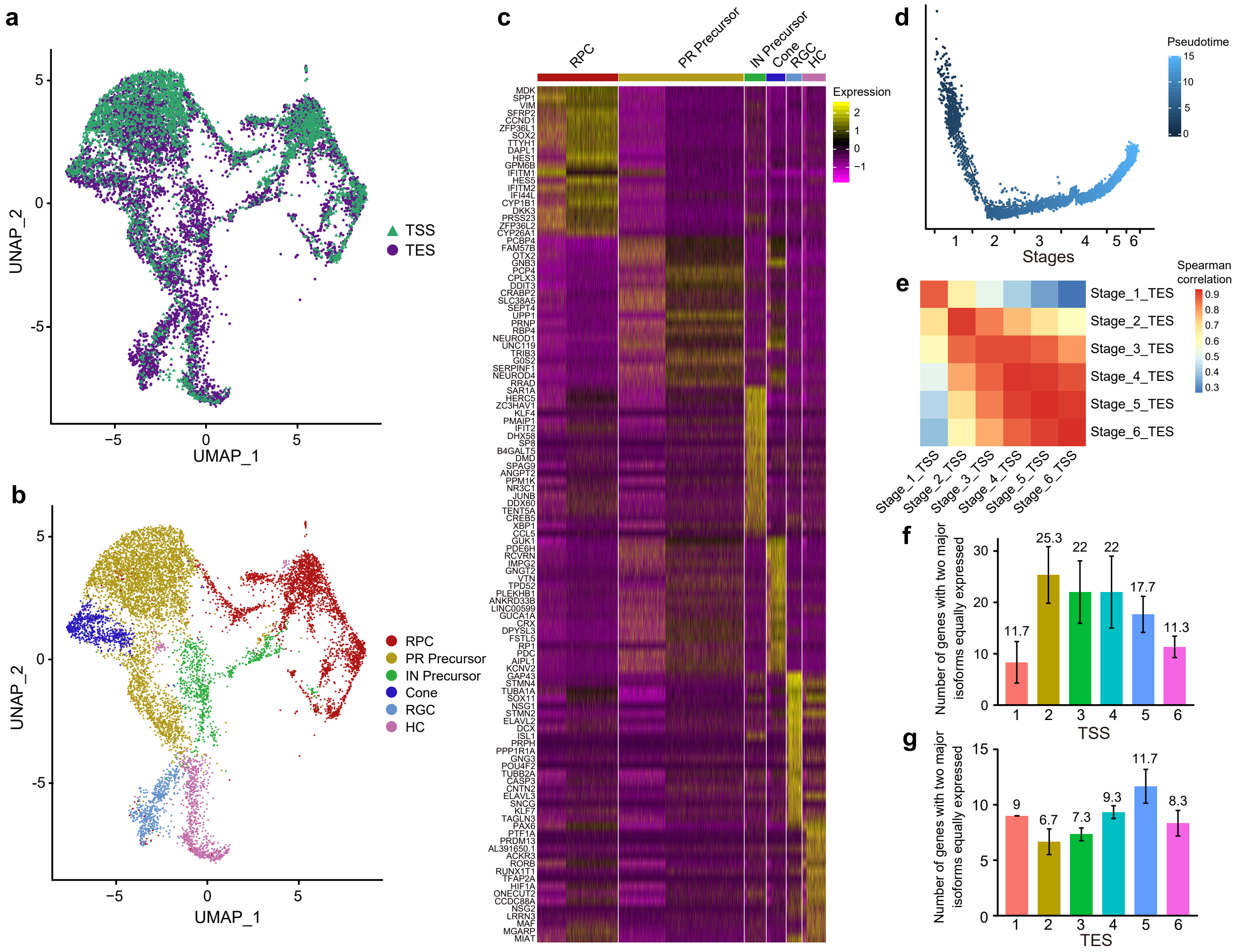

Supplementary Fig. 8. Dynamics for isoform choices during human cone development. a UMAP plot showing the distribution of the 5'-TSS and 3'-TES single cell data across the subtypes. **b** UMAP plot depicting cell clusters identified with scRCAT-seq, including RPC, PR Precursor, cone, Interneuron precursor (IN Precursor), Retinal ganglion cell (RGC), Horizontal cell (HC). **c** Heatmap showing the top 20 genes significantly enriched in each subcluster. **d** A scatterplot of pseudotime trajectory for RPC, PR precursor and Cone. Each dot represent a single cell and is colored according to the pseudotime value inferred. **e** Heatmap showing the correlation between TSS data and TES data for subgroups of single cells at different stages. **f** Barplots showing the number of genes with two major isoforms, which differ in TSS choices, that are equally expressed at the 6 stages from RPC to Cone. Error bars represent standard deviation of the mean (n = 3). **g** Barplots showing the number of genes with two major isoforms, which differ in TES choices, that are equally expressed at the 6 stages from RPC to Cone. Error bars represent standard deviation of the mean (n = 3). Source data are provided as a Source Data file.

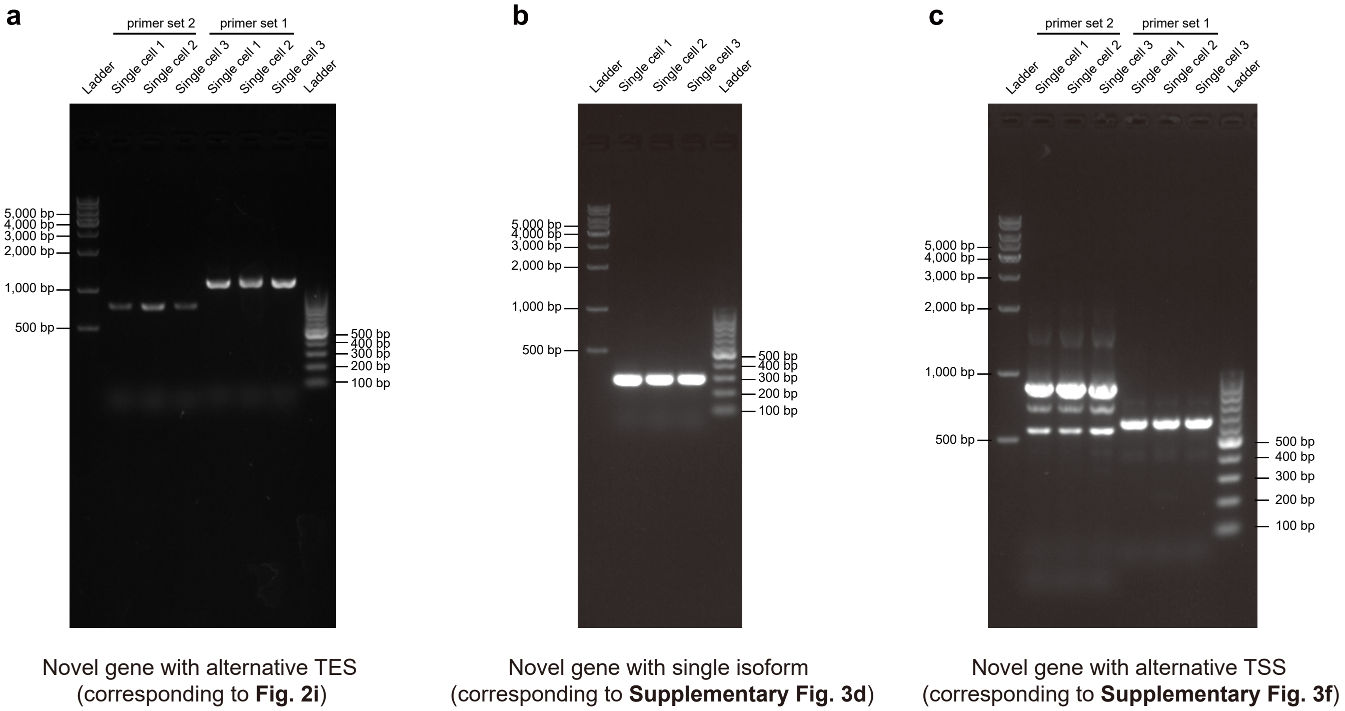

Supplementary Fig. 9. Uncropped gel images. a Uncropped gel image corresponding to Fig. 2i. **b** Uncropped gel image corresponding to Supplementary Fig. 3d. **c** Uncropped gel image corresponding to Supplementary Fig. 3f.

Supplementary Tables

Supplementary Table 1. Description of the ML features.

Apply to TES/TSS	Groups of the features	Name of the features	Description of the features
TES and TSS	Read distribution	RPM_of_peak	The total RPM value of the peak called by CAGEr.
TES and TSS	Read distribution	RPM_of_Dominant_Site	The highest RPM value of all sites within a peak.
TES and TSS	Read distribution	Peak_width	The width of the peak called by CAGEr.
TSS	Motif	BREu_motif_x (x=1, 2, ..., 50)	Whether there is BREu motif in x-nt upstream of TSS site
TSS	Motif	BREd_motif_x (x=1, 2, ..., 50)	Whether there is BREd motif in x-nt upstream of TSS site
TSS	Motif	TATA_motif_x (x=1, 2, ..., 50)	Whether there is TATA motif in x-nt upstream of TSS site
TES	Motif	AATAAA_x (x=1, 2, ..., 50)	Whether there is AATAAA motif in x-nt downstream of TSS site
TES	Motif	ATTAAA_x (x=1, 2, ..., 50)	Whether there is ATTAAA motif in x-nt downstream of TSS site
TES	Motif	AAGAAA_x (x=1, 2, ..., 50)	Whether there is AAGAAA motif in x-nt downstream of TSS site
TES	Motif	AATAGA_x (x=1, 2, ..., 50)	Whether there is AATAGA motif in x-nt downstream of TSS site
TES	Motif	AATACA_x (x=1, 2, ..., 50)	Whether there is AATACA motif in x-nt downstream of TSS site
TES	Motif	AATATA_x (x=1, 2, ..., 50)	Whether there is AATATA motif in x-nt downstream of TSS site
TES	Motif	AATGAA_x (x=1, 2, ..., 50)	Whether there is AATGAA motif in x-nt downstream of TSS site
TES	Motif	AGTAAA_x (x=1, 2, ..., 50)	Whether there is AGTAAA motif in x-nt downstream of TSS site
TES	Motif	ACTAAA_x (x=1, 2, ..., 50)	Whether there is ACTAAA motif in x-nt downstream of TSS site
TES	Motif	GATAAA_x (x=1, 2, ..., 50)	Whether there is GATAAA motif in x-nt downstream of TSS site
TES	Motif	CATAAA_x (x=1, 2, ..., 50)	Whether there is CATAAA motif in x-nt downstream of TSS site
TES	Motif	TATAAA_x (x=1, 2, ..., 50)	Whether there is TATAAA motif in x-nt downstream of TSS site
TES	Motif	TTTAAA_x (x=1, 2, ..., 50)	Whether there is TTTAAA motif in x-nt downstream of TSS site
TSS	Internal priming sites	3_G_3	3 Gs in 3-nt window upstream the TSS peaks.
TSS	Internal priming sites	3_G_2	≥ 2 Gs in 3-nt window upstream the TSS peaks
TSS	Internal priming sites	5_G_3	≥ 3 consecutive Gs in 5-nt window upstream the TSS peaks
TSS	Internal priming sites	5_G_4	≥ 4 Gs in 5-nt window upstream the TSS peaks
TSS	Internal priming sites	G_percentage_3	The percentage of Gs in 3-nt window upstream the TSS peaks
TES	Internal priming sites	5A_in_5	5 As in 5-nt window downstream the TES peaks
TES	Internal priming sites	6consecutiveA_in_10	≥ 6 consecutive As in 10-nt window downstream the TES peaks
TES	Internal priming sites	7A_in_10	≥ 7 As in 10-nt window downstream the TES peaks
TES	Internal priming sites	8A_in_10	≥ 8 As in 10-nt window downstream the TES peaks
TES	Internal priming sites	12A_in_15	≥ 12 As in 15-nt window downstream the TES peaks
TES	Internal priming sites	15A_in_20	≥ 15 As in 20-nt window downstream the TES peaks
TES	Internal priming sites	27AT_in_30	≥ 27 A/Ts in 30-nt window downstream the TES peaks
TES	Internal priming sites	33A_in_50	≥ 33 As in 50-nt window downstream the TES peaks
TES	Internal priming sites	A_percentage_5	The percentage of As in 5-nt window downstream the TES peaks
TES	Internal priming sites	A_percentage_10	The percentage of As in 10-nt window downstream the TES peaks
TES	Internal priming sites	A_percentage_15	The percentage of As in 15-nt window downstream the TES peaks
TES	Internal priming sites	A_percentage_20	The percentage of As in 20-nt window downstream the TES peaks
TES	Internal priming sites	A_percentage_30	The percentage of As in 30-nt window downstream the TES peaks
TES	Internal priming sites	A_percentage_50	The percentage of As in 50-nt window downstream the TES peaks

Supplementary Table 2. Sequencing depths of ERCC spike-in libraries

Sample	Sequencing depth
ERCC_01	1,052,270
ERCC_02	1,571,949
ERCC_03	3,799,426
ERCC_04	3,989,246
ERCC_05	2,291,544
ERCC_06	3,835,792
ERCC_07	3,986,186
ERCC_08	7,964,775
ERCC_09	7,645,733
ERCC_10	5,223,629
Average	4,136,055

Sequencing depth for each ERCC spike-in library generated by scRCAT-seq is listed.

Supplementary Table 3. Enriched motifs within 100bp of TSS peaks.

Distance to annotated TSS	Motif		P-value
10~100 bp	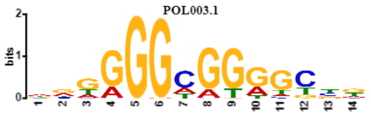	GC-box	1.72e-04
	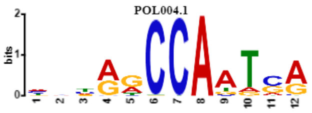	CCAAT-box	1.12e-02
100~500 bp	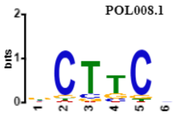	DCE1	1.24e-02
	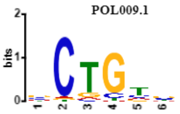	DCE2	9.04e-03
	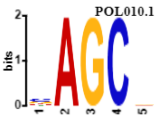	DCE3	5.83e-03
500~1000 bp	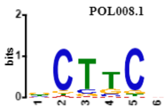	DCE1	2.69e-02
	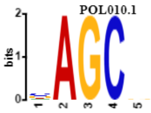	DCE3	3.87e-03
>1000 bp	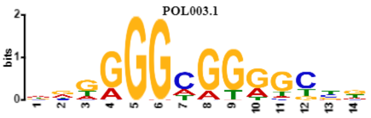	GC-box	3.06e-03
	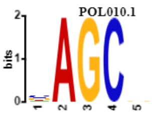	DCE3	3.87e-03

Enriched motifs within 100bp of TSS peaks are listed. Significance were computed using Tomtom[2].

Supplementary Table 4. Enriched motifs within 100bp of TES peaks.

Distance to annotated TES	Motif		p-value
10~100 bp	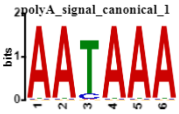	canonical polyA signal 1(AATAAA)	1.21e-02
	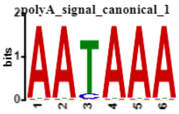	canonical polyA signal 1(AATAAA)	1.21e-02
100~500 bp	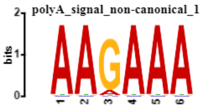	non-canonical polyA signal 1(AAGAAA)	1.93e-02
	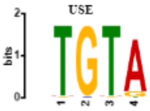	USE(TGTA)	7.34e-03
500~1000 bp	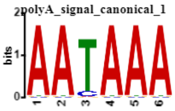	canonical polyA signal 1(AATAAA)	1.21e-02
	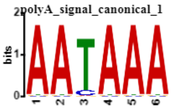	canonical polyA signal 1(AATAAA)	1.21e-02
>1000 bp	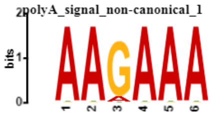	non-canonical polyA signal 1(AAGAAA)	3.39e-03
	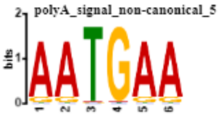	non-canonical polyA signal 5(AATGAA)	1.34e-02
	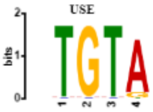	USE(TGTA)	1.43e-02

Enriched motifs within 100bp of TSS peaks are listed. Significance were computed using Tomtom[2].

Supplementary Table 5. Single-cell samples sequenced by scRCAT-seq.

Cell	Cell Num.	Methods	Library	Mapped reads	Transcripts	Acc.	Novel genes
DRG	18	Low throughput scRCAT-seq	TSS	17,413,957	17,563	79.0%	217
DRG	18	Low throughput scRCAT-seq	TES	2,698,441	23,619	87.8%	217
HEK293T	24	Low throughput scRCAT-seq	TSS	3,983,237	16,659	81.4%	45
HEK293T	24	Low throughput scRCAT-seq	TES	28,516,176	27,293	82.5%	45
hESC	23	Low throughput scRCAT-seq	TSS	6,696,786	17,482	83.9%	76
hESC	23	Low throughput scRCAT-seq	TES	51,061,170	23,829	86.5%	76
oocyte	8	Low throughput scRCAT-seq	TSS	9,604,656	29,083	74.4%	752
oocyte	8	Low throughput scRCAT-seq	TES	1,965,225	31,270	77.3%	752
ARPE19	1,065	High throughput scRCAT-seq	TSS	25,183,405	44,599	78.2%	-
HEK293T	892	High throughput scRCAT-seq	TSS	37,252,084	65,723	78.1%	-
hESC	928	High throughput scRCAT-seq	TSS	36,510,241	65,897	80.0%	-
mESC	1,436	High throughput scRCAT-seq	TSS	10,987,712	61,234	76.4%	-
organoid	3,407	High throughput scRCAT-seq	TSS	28,447,860	53,571	79.6%	258
organoid	6,395	High throughput scRCAT-seq	TES	94,052,459	180,324	90.1%	258

Supplementary Table 6. Samples sequenced by ScISOr-Seq in this study.

Sample	Subreads base (G)	Number of Subreads	Average subreads length	Number of CCS	Number of FLNC
DRG_1	0.32	214,295	1,487	19,947	4,289
DRG_2	0.35	237,777	1,481	23,070	3,835
OC_1	0.89	612,741	1,446	47,152	8,171
OC_2	0.24	165,171	1,436	13,305	780
OC_3	1.03	703,398	1,462	54,258	22,549
OC_4	3.52	2,492,563	1,414	193,637	13,932
OC_5	0.22	145,585	1,483	14,857	1,009
OC_6	1.31	912,567	1,441	68,471	27,559

Information about 6 single oocytes and 2 single DRG neurons generated by ScISOr-Seq are listed.

Supplementary Table 7. Cloning primers used in this study

Target gene	Sequences (5’ → 3’)
Novel gene 1 F	CTGCATCAGCTTCTGTTTCCT
Novel gene 1 R	GCTTAACAGTTTCGGAGGGT
Novel gene 2-1 F	CACTCCTCCACGGCCTC
Novel gene 2-1 R	TTCTTTACAGATATTTAAGGCACCC
Novel gene 2-2 F	GCTGGTCACGGTTGTACCTT
Novel gene 2-2 R	ATCATGGGAAGGGCATGAGC
Novel gene 3-1 F	TTACATGCTCTGACTTGGGCT
Novel gene 3-1 R	GTGTGCTCTGGCTTGCCATT
Novel gene 3-2 F	AGCCAACTCTAAGATGGCACC
Novel gene 3-2 R	CTGAGCTTCGGTTTGGTGTG

Primer sequences used to clone full-length novel genes are listed.

Supplementary Reference:

1. Gupta, I. et al. Single-cell isoform RNA sequencing characterizes isoforms in thousands of cerebellar cells. *Nature biotechnology* **36**, 1197-1202 (2018).
2. Gupta, S., Stamatoyannopoulos, J.A., Bailey, T.L. & Noble, W.S. Quantifying similarity between motifs. *Genome biology* **8**, R24-R24 (2007).
