# Supplementary material for: Single-cell RNA cap and tail sequencing (scRCAT-seq) reveals subtype-specific isoforms differing in transcript demarcation
Source: Nat Commun. 2020 Oct 13;11:5148. doi: 10.1038/s41467-020-18976-7 (PMC7555861; doi:10.1038/s41467-020-18976-7)
Supplement: Supplementary file 2 — Reporting Summary [file 41467_2020_18976_MOESM2_ESM.pdf]

nature research

Corresponding author(s): Yizhi Liu, Youjin Hu

Last updated by author(s): Aug 26, 2020

Reporting Summary

Nature Research wishes to improve the reproducibility of the work that we publish. This form provides structure for consistency and transparency in reporting. For further information on Nature Research policies, see Authors & Referees and the Editorial Policy Checklist.

Statistics

For all statistical analyses, confirm that the following items are present in the figure legend, table legend, main text, or Methods section.

n/a	Confirmed	
☐	☒	The exact sample size (*n*) for each experimental group/condition, given as a discrete number and unit of measurement
☐	☒	A statement on whether measurements were taken from distinct samples or whether the same sample was measured repeatedly
☐	☒	The statistical test(s) used AND whether they are one- or two-sided<br>*Only common tests should be described solely by name; describe more complex techniques in the Methods section.*
☒	☐	A description of all covariates tested
☐	☒	A description of any assumptions or corrections, such as tests of normality and adjustment for multiple comparisons
☐	☒	A full description of the statistical parameters including central tendency (e.g. means) or other basic estimates (e.g. regression coefficient) AND variation (e.g. standard deviation) or associated estimates of uncertainty (e.g. confidence intervals)
☐	☒	For null hypothesis testing, the test statistic (e.g. *F*, *t*, *r*) with confidence intervals, effect sizes, degrees of freedom and *P* value noted<br>*Give P values as exact values whenever suitable.*
☒	☐	For Bayesian analysis, information on the choice of priors and Markov chain Monte Carlo settings
☒	☐	For hierarchical and complex designs, identification of the appropriate level for tests and full reporting of outcomes
☐	☒	Estimates of effect sizes (e.g. Cohen's *d*, Pearson's *r*), indicating how they were calculated

Our web collection on statistics for biologists contains articles on many of the points above.

Software and code

Policy information about availability of computer code

Data collection	Bash, Python (version 3.7), R (version 3.6.0), Cell Ranger (version 3.1.0)
Data analysis	For data analysis, the following softwares were used, as described in the methods section: Python (version 3.7), R (version 3.6.0), STAR (version 2.7.3a), cutadapt (version 1.18), HTSeq (version 0.11.2), SCDE (version 2.10.1), BEDtools (version 2.27.1), Minimap2 (version 2.17), CAGEr (version 1.24.0), DESeq2 (version 1.26.0), Cell Ranger (version 3.1.0), Seurat (version 3.1.0), Monocle2 (version 2.12.0), Sequel SMRT-Link (version 7.0), cDNA_Cupcake (verision 12.4.0), SQANTI2 (version 7.4.0). The scRCAT-seq analysis pipeline is available at: https://github.com/huyoujinlab/scRCAT-seq.

For manuscripts utilizing custom algorithms or software that are central to the research but not yet described in published literature, software must be made available to editors/reviewers. We strongly encourage code deposition in a community repository (e.g. GitHub). See the Nature Research guidelines for submitting code & software for further information.

Data

Policy information about availability of data

All manuscripts must include a data availability statement. This statement should provide the following information, where applicable:

- Accession codes, unique identifiers, or web links for publicly available datasets
- A list of figures that have associated raw data
- A description of any restrictions on data availability

All sequencing data generated in this study has been uploaded at Gene Expression Omnibus (GEO) with accession number: GSE134311.
C1 CAGE data were downloaded from DDBJ (Project ID: PRJDB5282). C1 STRT data were downloaded from GEO(accession number: GSE60361). BAT-seq data were downloaded from GEO(accession number: GSE60768).
FANTOM5 annotations of hESC, HEK293T, mESC, ARPE, human retina, mouse dorsal root of spinal cord and mouse ovarium were download from https://fantom.gsc.riken.jp/5/datafiles/reprocessed/
PolyA_DB3 annotation of human and mouse were download from https://exon.apps.wistar.org/PolyA_DB/v3/

Field-specific reporting

Please select the one below that is the best fit for your research. If you are not sure, read the appropriate sections before making your selection.

☒ Life sciences ☐ Behavioural & social sciences ☐ Ecological, evolutionary & environmental sciences

For a reference copy of the document with all sections, see nature.com/documents/nr-reporting-summary-flat.pdf

Life sciences study design

All studies must disclose on these points even when the disclosure is negative.

Sample size	Sample sizes were not predetermined using statistical analysis, but for all biological samples analyzed, at least two biological replicates were used as the minimum required for comparison of the methods reproducibility. For 10x scRNA-seq, 14,196 cells were analyzed. This was not pre-determined or calculated.
Data exclusions	No data was specifically excluded, but filtering was performed as described in the Methods section using distclu peak calling and machine learning model to exclude peaks not likely to be derived from true 5' ends or 3' ends.
Replication	At least 2 biological replicates were profiled. Most reads are located in 5' end or 3' end (Fig. 2b and Supplementary Fig. 2c, n = 10 biological replicates), indicating high reproducibility of our methods. We validated scRCAT-seq identified novel genes by sequencing matched cDNA libraries on Sanger sequencing.
Randomization	4-6 week old C57BL/6 mice were randomly picked and allocated into experimental groups. Single DRG neurons were randomly picked with almost equal number of small, medium and large diameter neurons in order to incorporate most subtypes. Oocytes, HEK293T cells and hESC cells were also randomly picked.
Blinding	Yuanhui Qiu, the research assistant who performed scRCAT-seq and Smart-seq2 libraries construction, was blinded from any further details when picking individual cells to construct libraries.

Reporting for specific materials, systems and methods

We require information from authors about some types of materials, experimental systems and methods used in many studies. Here, indicate whether each material, system or method listed is relevant to your study. If you are not sure if a list item applies to your research, read the appropriate section before selecting a response.

Materials & experimental systems

n/a	Involved in the study	
☒	☐	Antibodies
☐	☒	Eukaryotic cell lines
☒	☐	Palaeontology
☐	☒	Animals and other organisms
☒	☐	Human research participants
☒	☐	Clinical data

Methods

n/a	Involved in the study	
☒	☐	ChIP-seq
☒	☐	Flow cytometry
☒	☐	MRI-based neuroimaging

Eukaryotic cell lines

Policy information about cell lines

Cell line source(s)	ARPE19(ATCC No. CRL-2302), HEK293T(ATCC No. CRL-3216) , hESC(H9)(ATCC No. HTB-176), mESC(E14Tg2a)(ATCC No. CRL-1821)
Authentication	Cell lines were not authenticated.
Mycoplasma contamination	Transcriptome alignment of the all sequencing data from all cell lines against 79 reference genomes of Mycoplasma or Acholeplasma, including Mycoplasma hominis, confirmed the absence of contamination.
Commonly misidentified lines (See ICLAC register)	No commonly misidentified cell lines were used in the study.

Animals and other organisms

Policy information about studies involving animals; ARRIVE guidelines recommended for reporting animal research

Laboratory animals	4-6 week old C57BL/6 mice of both genders were purchased from the Shanghai Model Organisms Center. Mice were maintained

Laboratory animals	under standard conditions (12 h light and dark cycles,room temperatures of 18-23°C , 40-60% humidity, with sufficient food and water).
Wild animals	Study did not involve wild animals.
Field-collected samples	Study did not involve field collected samples.
Ethics oversight	All animal procedures were conducted in an approval of the Institutional Animal Care and Use Committee (IACUC) of the Zhongshan Ophthalmic Center of Sun Yat-sen University (2018-171).

Note that full information on the approval of the study protocol must also be provided in the manuscript.
