# Supplementary material for: Single-cell RNA cap and tail sequencing (scRCAT-seq) reveals subtype-specific isoforms differing in transcript demarcation
Source: Nat Commun. 2020 Oct 13;11:5148. doi: 10.1038/s41467-020-18976-7 (PMC7555861; doi:10.1038/s41467-020-18976-7)
Supplement: Supplementary file 3 — Description of Additional Supplementary Files [file 41467_2020_18976_MOESM3_ESM.pdf]

**Description of Additional Supplementary Files**

File Name: Supplementary Software 1
Description: The pipeline for processing scRCAT-seq data. This pipeline is used to identify TSSs/TESs. The [output] and [output_customized] directories contain the results generated by the pipeline, including novel TSSs/TESs, the number of alternative TSSs/TESs and major TSS/TES of each gene. The [README.txt] file contains step-by-step instructions to implement. The [install] directory contains files to install required software and packages. The [script] and [bin] directories contain all needed scripts. The details of the pipeline can be found at https://github.com/huyoujinlab/scRCAT-seq.
