# Supplementary figures and images for: Single-cell RNA cap and tail sequencing (scRCAT-seq) reveals subtype-specific isoforms differing in transcript demarcation
Source: Nat Commun. 2020 Oct 13;11:5148. doi: 10.1038/s41467-020-18976-7 (PMC7555861; doi:10.1038/s41467-020-18976-7)

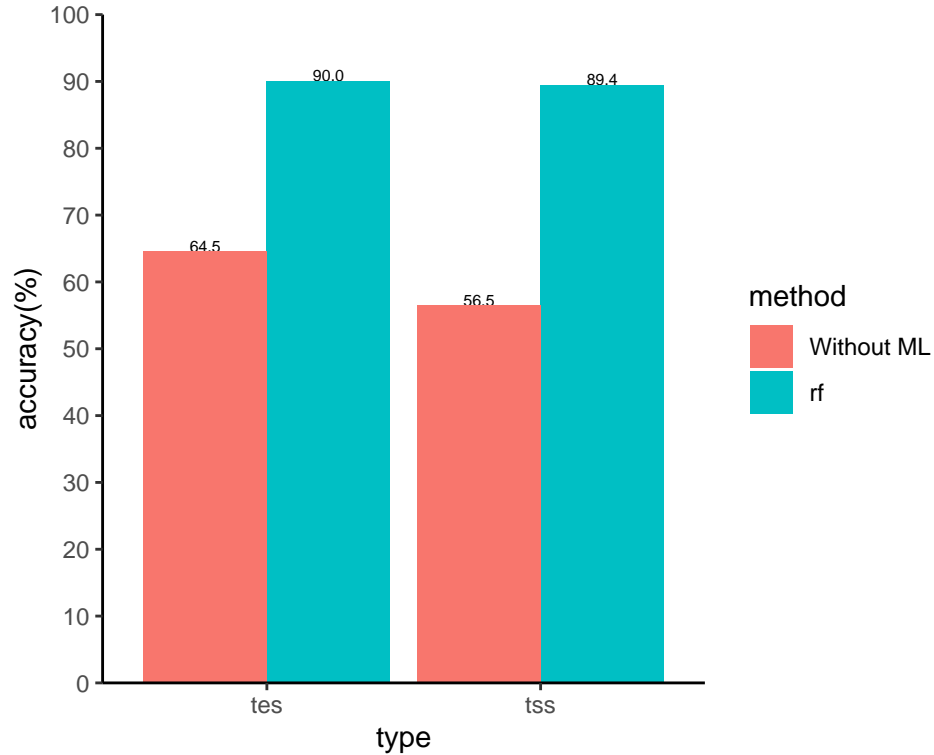

Supplement: Supplementary file 4 — Supplementary Software 1 [file 41467_2020_18976_MOESM4_ESM.zip › Supplementary Software/software_checklist/output/accuracy.pdf]

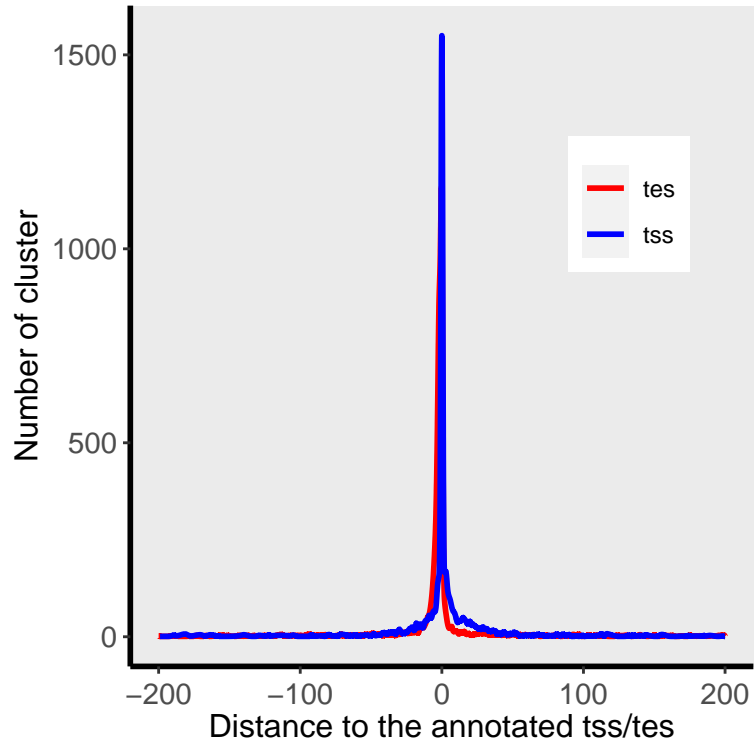

Supplement: Supplementary file 4 — Supplementary Software 1 [file 41467_2020_18976_MOESM4_ESM.zip › Supplementary Software/software_checklist/output/distance.pdf]

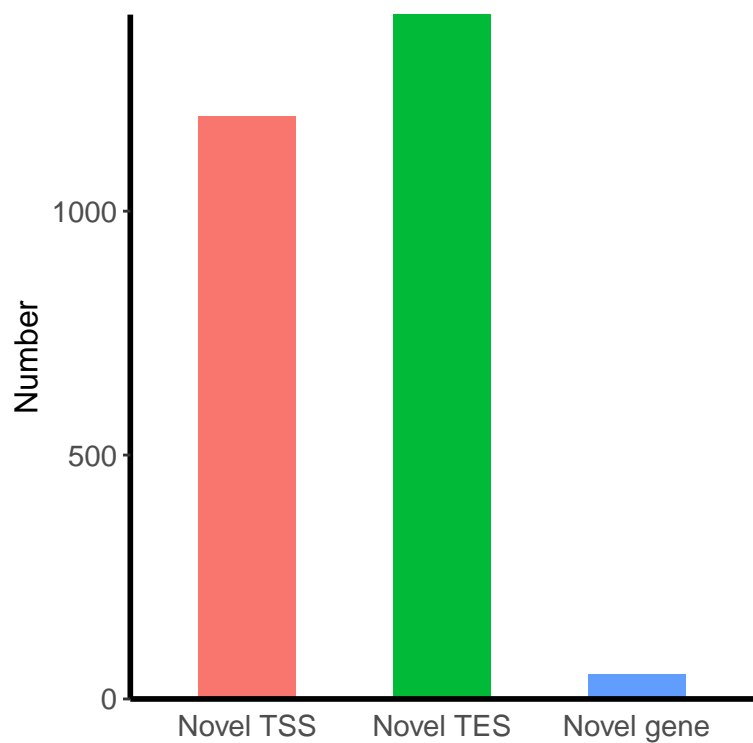

Supplement: Supplementary file 4 — Supplementary Software 1 [file 41467_2020_18976_MOESM4_ESM.zip › Supplementary Software/software_checklist/output/novel.pdf]

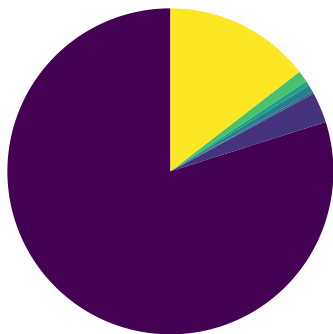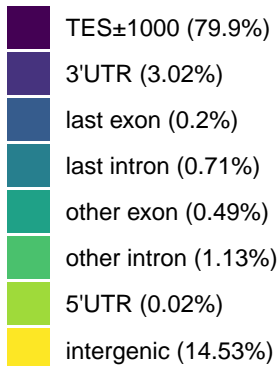

Supplement: Supplementary file 4 — Supplementary Software 1 [file 41467_2020_18976_MOESM4_ESM.zip › Supplementary Software/software_checklist/output/pieTES.pdf]

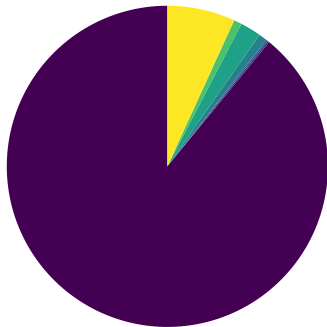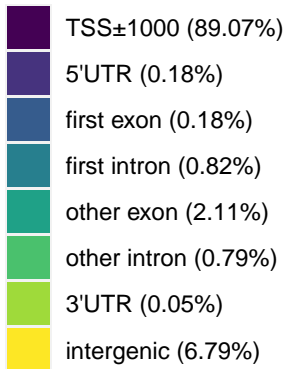

Supplement: Supplementary file 4 — Supplementary Software 1 [file 41467_2020_18976_MOESM4_ESM.zip › Supplementary Software/software_checklist/output/pieTSS.pdf]
